# Glioblastoma CD105^+^ cells define a SOX2^−^ cancer stem cell-like subpopulation in the pre-invasive niche

**DOI:** 10.1186/s40478-022-01422-8

**Published:** 2022-08-29

**Authors:** Jiaxin Li, Fredrik Ek, Roger Olsson, Mattias Belting, Johan Bengzon

**Affiliations:** 1 Stem Cell Center, Lund University, Lund, Sweden; 2 Division of Neurosurgery, Department of Clinical Sciences, Lund University, Lund, Sweden; 3 Department of Neurosurgery, Skane University Hospital, Lund, Sweden; 4 Chemical Biology and Therapeutics, Department of Experimental Medical Science, Lund University, Lund, Sweden; 5 Section of Oncology, Department of Clinical Sciences, Lund University, Lund, Sweden; 6 Department of Hematology, Oncology and Radiophysics, Skane University Hospital, Lund, Sweden; 7 Science for Life Laboratory, Department of Immunology, Genetics, and Pathology, Uppsala University, Uppsala, Sweden

**Keywords:** CD105, Glioma stem-like cell, Exome sequencing, Drug screening, Tumor microenvironment

## Abstract

Glioblastoma (GBM) is the most common and most aggressive primary brain tumor in adults. Glioma stem like cells (GSC) represent the highest cellular hierarchy in GBM and have a determining role in tumor growth, recurrence and patient prognosis. However, a better definition of GSC subpopulations, especially at the surgical resection margin, is warranted for improved oncological treatment options. The present study interrogated cells expressing CD105 (CD105^+^) specifically within the tumor front and the pre-invasive niche as a potential GSC subpopulation. GBM primary cell lines were generated from patients (n = 18) and CD105^+^ cells were isolated and assessed for stem-like characteristics. In vitro, CD105^+^ cells proliferated and enriched in serum-containing medium but not in serum-free conditions. CD105^+^ cells were characterized by Nestin^+^, Vimentin^+^ and SOX2^−^, clearly distinguishing them from SOX2^+^ GCS. GBM CD105^+^ cells differentiated into osteocytes and adipocytes but not chondrocytes. Exome sequencing revealed that GBM CD105^+^ cells matched 83% of somatic mutations in the Cancer cell line encyclopedia, indicating a malignant phenotype and in vivo xenotransplantation assays verified their tumorigenic potential. Cytokine assays showed that immunosuppressive and protumorigenic cytokines such as IL6, IL8, CCL2, CXCL-1 were produced by CD105^+^ cells. Finally, screening for 88 clinical drugs revealed that GBM CD105^+^ cells are resistant to most chemotherapeutics except Doxorubicin, Idarubicin, Fludarabine and ABT-751. Our study provides a rationale for targeting tumoral CD105^+^ cells in order to reshape the tumor microenvironment and block GBM progression.

**Supplementary Information:**

The online version contains supplementary material available at 10.1186/s40478-022-01422-8.

## Introduction

Patients suffering from the most aggressive primary brain tumors, glioblastoma (GBM), have a short survival despite extensive surgical resection and subsequent chemo- and radiation therapy [1–3]. Unfortunately, tumor recurrence occurs almost without exception. Treatment failure is multifaceted, but a major contributing factor is the presence of subpopulations of glioma stem-like cells (GSC) [4, 5]. Glioma stem like cells represent the highest cellular hierarchy in GBM and these cells proliferate, self-renew and generate heterogenous clones that comprise the bulk of the tumor [6]. Moreover, GSC resists conventional radiotherapy and chemotherapy through their relative quiescence and plasticity [7]. Therefore, targeting GSC through identified cell surface markers has become a significant focus and this approach has showed promise to eliminate neoplastic progression in preclinical settings [8]. However, most GSC targeted therapies failed in GBM clinical trials [8, 9] and the major hypothesized explanation is the existence of multiple out-of-target GSC subpopulations. Thus, failure of anti-GSC therapies is related to unclear basic characterization of each GSC population. Exploring and characterizing putative new GSC subpopulations will possibly provide direct translational implications for GBM treatment.

CD105/Endoglin, a type I transmembrane protein belonging to the transforming growth factor (TGF) beta receptor family, is regarded as a cancer angiogenic marker [10, 11]. Its expression correlates with tumor progression, metastasis and poor prognosis in several solid cancers including GBM [12]. Moreover, CD105 is abundantly expressed on M2 tumor associated macrophages (TAMs), cancer associated fibroblasts (CAFs), Treg cells, and mesenchymal stem cells (MSCs) [13, 14], indicating that CD105 may play a central role in the generation of an immune suppressive tumor microenvironment (TME). In the stem cell context, CD105 is verified as a specific cell surface marker in both MSCs and cancer stem cells (CSC) [15]. In GBM, numerous studies have shown robust CD105 expression on tumor proliferative blood vessels and no or very low expression in the normal brain. Therefore, CD105 has been thoroughly investigated as an antiangiogenic therapeutic target for several years [16]. However, antibody-based therapies, which showed marked anti-tumoral effect against GBM in preclinical studies, subsequently proved unsuccessful in GBM patients [17].

Abnormal angiogenic networks are abundant in the actively growing GBM tumor front. In preoperative MRI, the tumor front is identified as the gadolinium-enhanced area and regarded as a tumor margin during operative resection [18]. However, even after complete resection of the gadolinium-enhanced regions, 85% of GBM recurrences will occur in the vicinity of the resection border [19, 20]. Thus, GSCs outside of the resected tumor margin may likely exist and these cells may be crucial for tumor recurrence. To clarify the existence of GSCs outside the tumor front, we obtained biopsies from GBM patients in the immediate peri-tumor region and mapped the tumor stem-like cells with the markers Sox2, Nestin and CD105. Both Sox2^+^Nestin^+^ and CD105^+^Nestin^+^ cell populations exist in this area, but CD105^+^Nestin^+^ cells were more numerous and locate around angiogenic tumor capillaries. Based on this preliminary clinical finding, we hypothesized that CD105^+^Nestin^+^ cells outside the tumor front represent a subset of GSCs and using fresh tissue from tumor and peri-tumor regions, we purified the CD105^+^ cells by fluorescence-activated cell sorting (FACS) and characterized them. Exome sequencing and in vivo xenografting were performed to assess tumorigenic mutations and tumorigenicity. In vitro GBM-derived CD105^+^ cell production of extracellular cytokines was measured to clarify possible cross talk between and the TME. Drug sensitivity screening was conducted to identify potential specific chemotherapeutics against CD105^+^ cells. We identified CD105^+^ cells as a new putative GSC subpopulation in the GBM pre-invasive niche.

## Materials and methods

### Tumor samples

Resected tissue from 18 GBM patients (Additional file 1: Table S1) was collected from the neurosurgery department at Skane University Hospital in Lund, Sweden. Written consent was signed by each patient in accordance with the approved ethical permit from the regional Swedish ethics committee (Dnr 2018/37). Using a surgical navigation system (Medtronic, USA), peri-tumor tissue was resected according to the treatment plan and tumor tissue was obtained. Anatomical, pathological diagnosis and routine molecular analysis were ascertained in each case by Skane University Hospital pathology department according to WHO GBM criteria. The resected tissue was divided into two parts: one part was fixed with 4% Paraformaldehyde (PFA) for tissue staining and the other part was put into ice-cold artificial CSF [21] for primary cell culture.

### Cell culture

Fresh surgical tissue in artificial CSF was immediately transferred to the lab and dissociated as a cell suspension. Dissociated cells were divided into two parts and grown in different conditions: serum-free condition (SFC), i. e. Neurobasal medium (Gibco) added with 1 mM sodium pyruvate (Gibco), 1 × B-27 supplement (Gibco), 1 × N-2 supplement (Gibco), 1 × non-essential amino acids (NEEA) (Gibco) or serum-condition (SC) DMEM/F12 (Gibco) supplemented with 10% fetal bovine serum (FBS) (Gibco). Cells in SFC were supplemented with EGF (Thermo Fisher Scientific) and FGF (Thermo Fisher Scientific) 20 ng/ml and grown on the Poly-l-ornithine (Merck) and Laminin (Gibco) coated dishes.

The U87 cell line was maintained and expanded in DMEM medium (Gibco) supplemented with 1% NEAA and 10% FBS. The GL261 mouse glioma cell line was maintained in RPMI 1640 medium (Gibco) supplemented with 1 mM sodium pyruvate and 10% FBS. Bone marrow-derived mesenchymal stromal cells (BM-MSCs) were collected from healthy donors as described previously [22]. BM-MSCs were maintained in StemMACS MSC Expansion Media (Miltenyi Biotec) added with 10% FBS and L-glutamine (Gibco). All the media were supplemented with 1% Penicillin–Streptomycin (Gibco) and mycoplasma detection was performed using MycoProbe Mycoplasma Detection Kit (R&D). Primary cells were expanded in culture up to passage five and then frozen at − 150 °C.

### Flow cytometry

Primary cells were detached by incubating with Accutase (Gibco) at 37 °C for 4 min. After dissociation, cells were diluted with FACS buffer (eBioscience) and filtered through a 75 µm cell strainer (Fisher Scientific) to obtain a single cell suspension. Cells were counted and approximately 1 million cells were aliquoted into each sterile Eppendorf tube. Samples were stained with the following antibodies: PE Mouse anti-Human CD105 Clone 266 (RUO) (BD Biosciences), Human Endoglin/CD105 PE-conjugated Antibody (R&D), APC-R700 Mouse Anti-Human CD274 Clone MIH1 (RUO) (BD Biosciences), APC Mouse Anti-Human CD133 Clone W6B3C1 (BD Biosciences), APC-R700 Mouse IgG1, κ Isotype Control (BD Biosciences), PE Mouse IgG1, κ Isotype Control (BD Biosciences) and incubated on ice for 30 min. Samples were washed with FACS buffer for 3 times and 7-AAD (BD Biosciences) or Hoechst 33,342 (BD Biosciences) was added as cell viability markers. Cells were sorted on a FACSAria III cell sorter (BD Biosciences, USA). Data was analyzed by FLOWJO software (BD Biosciences, USA).

### Immunohistochemistry

Frozen tissue was cut into 10 µm thickness using a cryostat (Leica, Germany). Cells was seeded on 8 well CultureSlides (Falcon, USA) and fixed by 4% PFA. Samples were washed with Phosphate Buffered Saline (PBS) (Gibco) and incubated for 1 h with PBS buffer containing 0.25% Triton X-100 and 5% Bovine Serum Albumin (BSA) (Merck) or 5% Donkey serum (Jackson ImmunoResearch) or 5% Goat serum (Jackson ImmunoResearch). The following primary antibodies was used for incubating at 37 °C for 1 h or 4 °C overnight: CD105 (1:100, AF1097, R&D), CD105 (1:200, AF1320, R&D), PD-L1(1:100, R&D), hFABP4 (1:100, R&D), hOsteocalcin (1:100, R&D) and hAggrecan (1:100, R&D), CD31 (1:100, Dako), NG2 (R&D, 1:200), Vimentin (1:500, Dako), Hu-Nu (1:250, Merck), SOX2 (1:200, Merck), CD73 (1:100, Merck), α-SMA (1:250, Merck), CD68 (1:100, Gene Tex), CD163 (1:100, Gene Tex), Nestin (1:200, Abcam), β III Tubulin (1:500, Abcam), Ki67 (1:250, Abcam), Iba1 (1:200, Abcam), CD11b (1:200, Abcam), NeuN (1:200, Abcam), GFAP ( 1:1000, Abcam), S100β (1:200, Abcam), CD34 (1:200, Abcam), vWF (1:100, Abcam), FAP (1:100, Invitrogen), CD90 (1:100, Santa Cruz), PDGF-β (1:200, Santa Cruz). Secondary antibodies conjugated to DyLight 488, 594 and 647 (1:200, Jackson ImmunoResearch) were applied for 1 h incubation and nuclei were stained with DAPI (1:1000, Invitrogen). Images were captured by an epifluorescence Olympus BX61 Microscope equipped with an Olympus DP80 Color Camera—9MP and CellSens acquisition software (Olympus Sverige AB, Solna, Sweden), or a Zeiss LSM 780 confocal microscope and Zeiss ZEN software (Carl Zeiss Microscopy GmbH, Germany).

### Cell viability assay

Cell viability was detected by the PrestoBlue™ Cell Viability Reagent (Thermo Fisher Scientific) according to the manufacturer’s protocol. Briefly, 1000 cells were seeded in 96 microplate wells (Greiner Bio-One) with 90 µl cell culture media in each well. 10 µl PrestoBlue regent was added for 10 min at 37 °C. Fluorescence units were read by a fluorescence microplate reader (Molecular Devices, USA) at 590 nm emission.

### Mesenchymal stem cell assay

GBM CD105^+^ cells at low passage (P3-P5) were used for mesenchymal stem cell (MSC) differentiation assays using a human MSC identification kit (R&D) according to the protocol provided by the manufacturer. Briefly, GBM CD105^+^ cells were cultured in human/mouse StemXVivo Osteogenic/ Adipogenic Base Media (R&D) with Adipogenic Supplement(R&D) and Osteogenic Supplement(R&D), respectively, for up to 14 days for adipogenic di­fferentiation or osteogenic differentiation. For chondrogenic diff­erentiation, cells were culture in human StemXVivo Chondrogenic Media (R&D) supplemented with ITS Supplement (R&D) and pelleted in 5 ml tube for up to 20 days. Cells were fixed and detected by anti-hFABP4, anti-hOsteocalcin and anti-hAggrecan immunohistochemistry.

### GBM CD105^+^ spheroid formation

Twelve-well dishes were precoated with 0.01% Poly-L-ornithine overnight and washed twice with PBS before coating with 10 µl/ml laminin for 4 h. 0.5 × 10^6^ GBM CD105^+^ cells were seeded in DMEM/F12 medium supplemented with 10% FBS. b-FGF 20 ng/ml was added every 48 h and the medium was changed every 3 days. Cells were inspected daily using an inverted light microscope. Once spheres were formed, the culture was filtered through a 70 µm cell strainer and spheres transferred into a 5 ml tube with fresh medium. The tube was centrifuged at 200 × *g* to pellet the spheres. Spheres were then sectioned by the cryostat before staining.

### DNA isolation and library preparation

Genomic DNA was isolated from the cells using the QIAamp DNA Micro kit (Qiagen) according to the manufacturer's protocol. The isolated DNA was dissolved in 25 μl of EB buffer (Qiagen) and the quality quantity was detected by the Nanodrop ND 1000 spectrophotometer (Thermo Fisher Scientific, USA), and stored at -20 °C. To construct the Library, DNA was diluted to 10 ng/μl in a total volume of 50 μl with 1 × EB buffer. Adaptor-ligated libraries was set by unique dual indices using KAPA-HyperPrep-Kit (Roche). Fragmentation of DNA was accomplished by sonication using the following parameters: 75 s Peak Power, 50.0 Duty Factor, 10.0 Cycles and burst 1000. After End repair and A-Tailing, adapter ligation was performed using 2 µl X Gen Duplex Adapters (15 µM) 30 min at 20 °C. The adaptor-ligated library was PCR amplified by 5 cycles according to the manufacturer's instructions with 8-bp unique dual index primer pairs. Hybridization of samples targeting predetermined regions of the genome provided by Twist Target Enrichment Protocol (Twist Bioscience) using Twist Hybridization and Wash kit (Twist Bioscience). The probes are labeled with biotin and streptavidin-conjugated magnetic beads (Lablife Nordic) purified DNA libraries. The adaptor libraries contain both primer sites used for post capture amplification by PCR methods. The quality and quantity of the exon-enriched capture library was measured by Agilent 2100 Bioanalyzer (Agilent, USA).

### DNA sequencing and data analysis

The pooled libraries at 0.7 nM loading concentration and 1% of PhiX Control (Illumina) were sequenced in a NovaSeq 6000 System (Illumina, USA) using NovaSeq 6000 SP Reagent Kit (Illumina), following the manufacturer's instructions. The sequencing data was analyzed on Illumina DRAGEN Bio-IT Platform (Illumina, USA) with the following steps. Raw basecalls were converted to Fastq files. Adapters are trimmed if added to samplesheet. Fastqs were mapped and aligned to the reference genome (hg38) (http://www.ncbi.nlm.nih.gov). Variants (SNV and SV) were called in the target region. Clinical grade annotation of all variants was passed with basic filtering in the DRAGEN platform. The quality of metrics from raw sequencing reads was calculated by FastQC [23].

#### In vitro cytokine array

Cell culture medium from 1 × 10^6^ cells was collected after 72 h and centrifuged at 1000* g* for 5 min. The supernatant was aspirated and stored at − 80 °C. Cytokine array was performed using a Human Angiogenesis Array C1 kits (RayBiotech) according to the manufacturer's instructions. Culture medium with 10% FBS was used as control. The membrane was scanned by azure biosystems C600 (Azure Biosystems, USA), and protein levels were semi-quantified by measuring the gray level values using Image J software [24].

#### Temozolomide (TMZ) and bevacizumab resistant assays

1000 CD105^+^ cells per well were cultured in 96 Well plates (Greiner Bio-One). Cells were treated with different concentration of TMZ (0.1 µM to 50 µM) and bevacizumab (10–2000 ng/ml) and cocultured for up to 96 h. Cell viability was detected by PrestoBlue™ Cell Viability Reagent according to manufactures’ protocol at 24 h, 48 h and 96 h. Fresh drug and media were added after the detection. Data was analyzed by Soft Max Pro V6.4 (Molecular Devices, USA).

#### Small molecule screening and analysis

The 88 anticancer drugs were selected from Selleckchem (L3000) library. The cells were seeded in 96-well plates at 1000 cells per well. The chemical compounds were dissolved in DMSO with two concentrations (0.5 μM and 10 μM) and added to the previous 96-well plates. The same volumes of DMSO (0.005% and 0.1% in volume) were added to the control group. Cells were incubated in 5% CO2 at 37 °C for 96 h and the cell viability was detected at 48 h and 96 h. After the drug sequencing, the 4 most effective drugs were selected. A drug-gene interaction analysis of the effects of these compounds were predicted by the DGIdb database as previously described [25]. The relation between the 10 genes with the highest drug-gene interaction score and GBM patient survival (TCGA database) was analyzed on cBiopartia online platform (https://www.cbioportal.org).

#### Animal studies

All animal procedures were guided by the practices of the Swedish Board of Animal Research and approved by the Committee of Animal Ethics in Lund-Malmo, Sweden (permit 5372–20). The JAX®NSG® mouse model (Charles River) was a gift from Dr. Henrik Ahlenius. Female mice at 6 weeks were used for tumor cell injection. Mice were anesthetized with isoflurane and fixed in a stereotactic frame (David Kopf Instruments, CA). Cells were suspended in culture media at a concentration of 50,000 cells/µl and 5 µl was injected into the right striatal region using a Hamilton syringe (Hamilton, Switzerland). Immediately after the appearance of neurological symptoms, the brain was harvested following transcardial perfusion with 4% PFA. Tumor volumes were macroscopically assessed in coronal sections and calculated from the formula (length × width^2^)/2.

#### Statistical analysis

Statistical analyses were performed using GraphPad Prism (GraphPad Software Inc., CA). Results are presented as mean ± SD. Comparisons between groups were performed by two-tailed Student’s t test or by one-way ANOVA, followed by Tukey’s multiple comparisons test. Kaplan–Meier survival curves were compared using a log rank test. R Studio with R packages (http://www.rstudio.com) was used for statistical analysis of DNA sequencing and Drug sequencing. Survival curves were analyzed by the online platform: cBiopartial (https://www.cbioportal.org) and GEPIA2 (http://gepia2.cancer-pku.cn). *P* < 0.05 was considered statistically significant.

## Results

### CD105^+^Nestin^+^ cells within the peritumor vascular niche

**Fig. 1:**
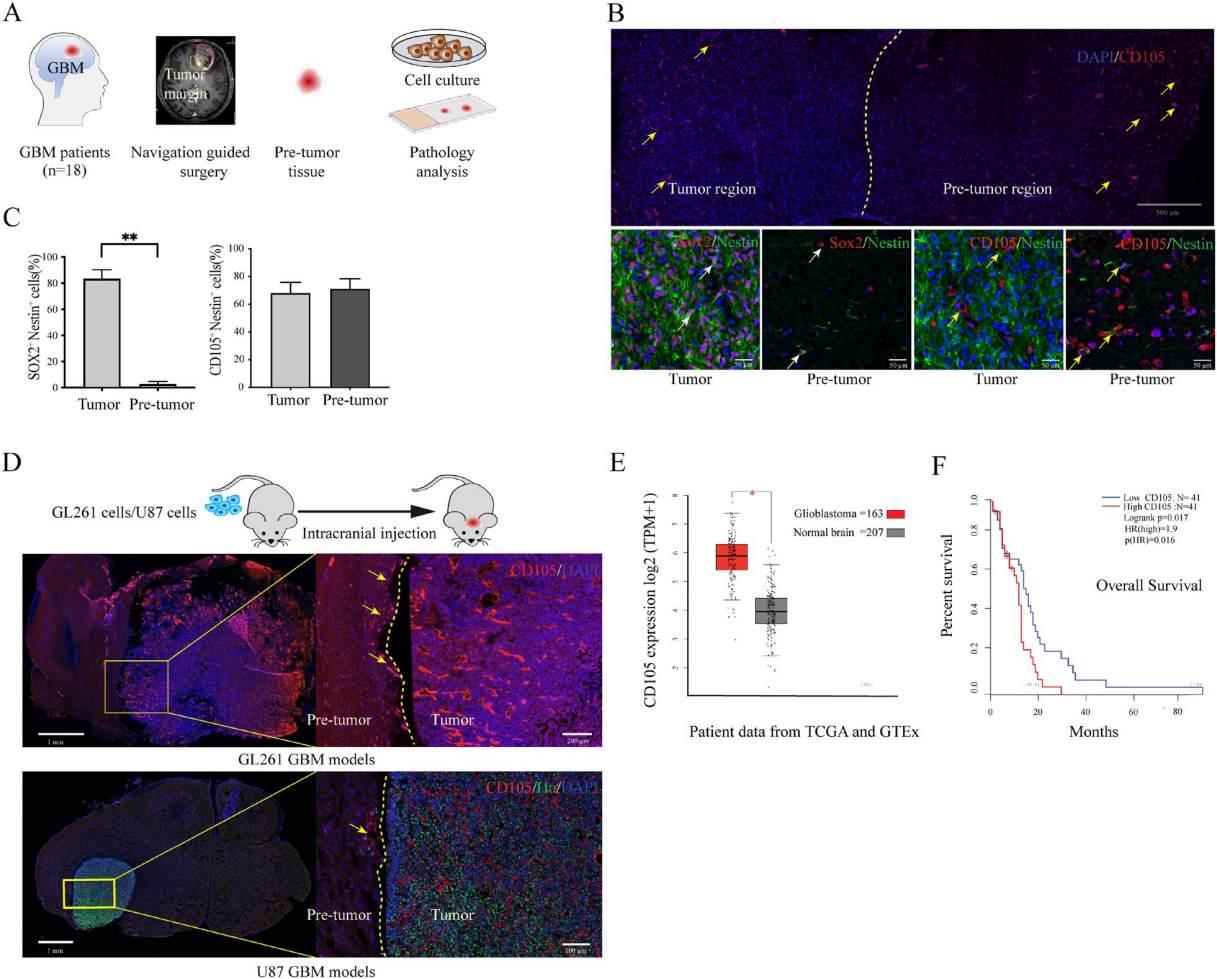
GBM CD105^+^ cell localization and specificity. **A** Schematic of clinical tissue collection. A total of 19 GBM border tissue samples were collected with guidance from the surgical navigation system. Each sample was split into two parts for different studies. **B** The landscape of CD105^+^ cells and cancer cells in GBM border tissue. Brain section containing the border between tumor and normal brain (pre-invasive niche) stained with CD105 showing the distribution of CD105^+^ cells. The border between tumor and peritumor area is identified by a difference in cell density (yellow line). High magnification images of tumor cells are shown using the markers CD105, SOX2 and Nestin. **C** Quantification of SOX2^+^ Nestin^+^ and CD105^+^ Nestin^+^ cells in tumor and peritumor tissue ** P < 0.01. **D** Evaluation of CD105^+^ cells in GBM in vivo models. Sections showing tumors at 25 days following intracranial injection of GL261 and U87 cells in mouse brains. Sections were stained with CD105 and Hu antibodies. Magnified images showing the border between tumor and brain. **E** Comparing the CD105 expression in 163 GBM samples from TCGA database and 207 normal brain samples from GTEx database *P < 0.05. **F** Overall survival correlated with CD105 RNA expression as analyzed from the data of 82 GBM patients in the TCGA database

Biopsies from the tumor border of GBM patients were obtained using MRI-based neuronavigation (Fig. 1A). Immunohistochemistry was performed using SOX2, Nestin and CD105 as markers to identify stem-like cells. DAPI and Ki67 staining depicted tumoral and peritumoral regions (Additional file 3: Fig S1A). SOX2^+^Nestin^+^ cells were abundant within the tumor boundary but only sporadically outside of the tumor. In contrast, numerous CD105^+^Nestin^+^ cells resided both within the tumor bulk and in the peritumor vascular structures (Fig. 1B, Additional file 3: Fig S1B). Quantification of cells revealed that SOX2^+^Nestin^+^ cells were significantly more abundant within the tumor bulk compared to the peritumor area (*P* = 0.01) whereas CD105^+^ Nestin^+^ cells were found dispersed without any significant difference in the density of distribution between the two regions (Fig. 1C). To detect whether CD105^+^ cells exist outside the tumor margin also in animal models, we used GL261 and U87 cell lines injected into the mouse brain. In both GBM models, immunofluorescence confirmed an abundance of CD105^+^ cells within the pre-invasive niche (Fig. 1D). To further evaluate expression of CD105 in human GBM, analysis of 163 GBM patients from the The Cancer Genome Atlas (TCGA) database (https://www.cancer.gov/about-nci/organization/ccg/research/structural-genomics/tcga) and 207 normal brain samples from Genotype-Tissue Expression (GTEx) database showed CD105 RNA expression to be significantly higher in GBM tissue compared to normal brain (*P* < 0.05) (Fig. 1E, Additional file 3: Fig S1C). Additionally, we investigated the relation between CD105 gene expression and clinical prognosis. GBM patients from the TCGA database were divided into quartiles based on levels of CD105 expression. The difference in overall survival between the lowest and highest quartile was significant (*P* = 0.016), indicating that high CD105 expression correlates with poor survival (Fig. 1F). In brief, CD105 cells are present in the preinvasive niche in GBM and expression levels correlate with patient survival.

### Primary CD105^+^ cell lines prefer serum condition in vitro

**Fig. 2:**
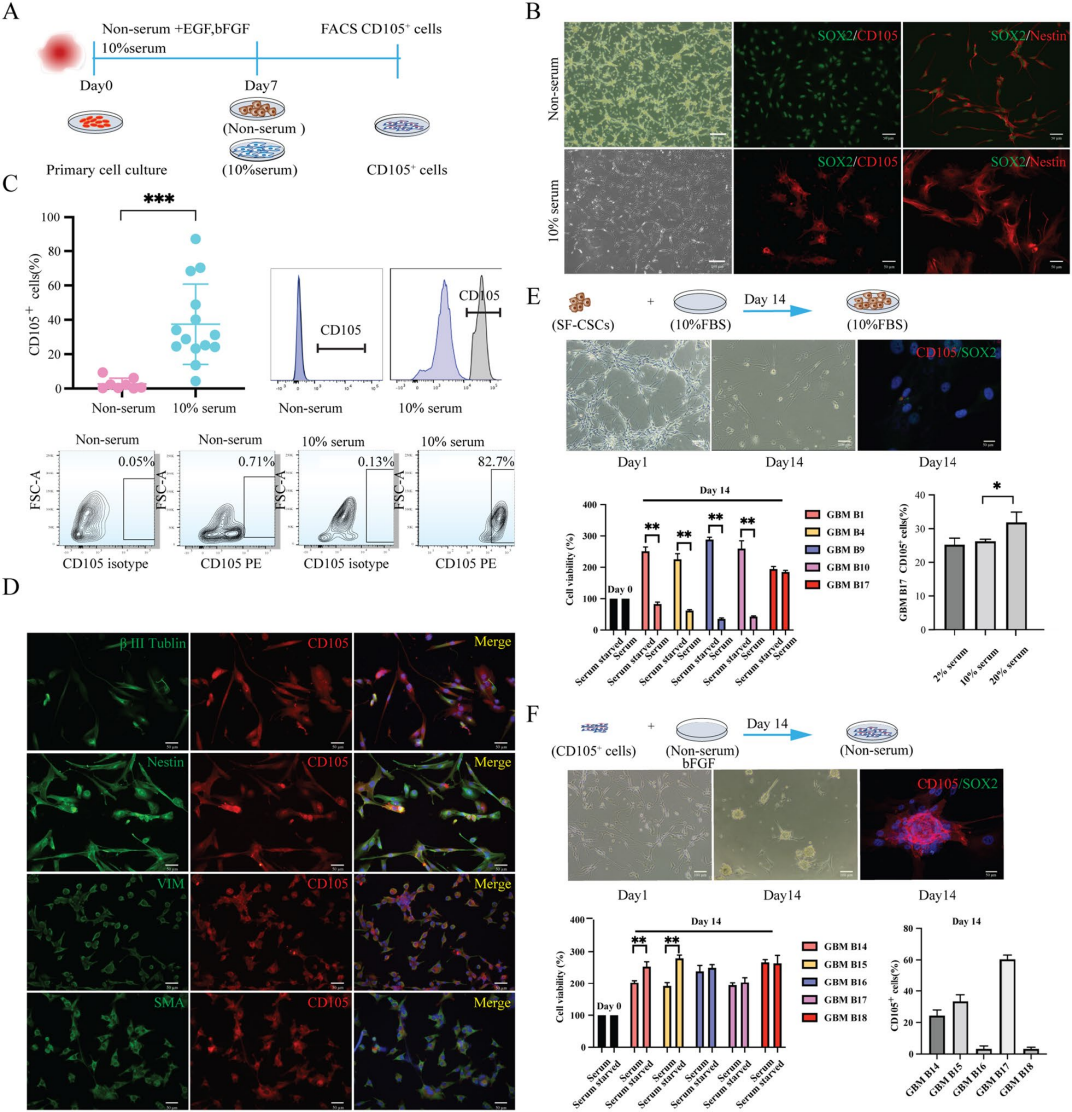
Culture conditions can interfere with the fate of GBM CD105^+^ cells. **A** Schematic representation of media conditions for CD105^+^ primary cell culture. **B** Primary cell culture in SC and SFC under passage 3 (P3). CD105^+^ cells and neural stem-like cells were identified by staining of CD105 (red) and SOX2 (green), respectively. **C** Flow cytometry analysis of CD105^+^ cells in different media conditions. Flow cytometry data show the GBM CD105 subpopulation obtained from GBM primary cells using the phycoerythrin (PE) channel. ***P < 0.001. **D** Immunostaining of CD105^+^ cell with cell type markers. Double immunostaining of CD105 (red) and cellular markers (green) on sorted CD105^+^ cells (under P3). **E**, **F** Effect of serum/ serum-free culture conditions on the differentiation of GBM primary cell lines. SFC cultured SOX2^+^ cells differentiated into DMEM/F12 media supplemented with 10% FBS for 14 days (**E**). Immunostaining of CD105 (red) and SOX2 (green) shown on differentiated cells (DAPI blue). Bar graph showing cell viability of 5 GBM SOX2^+^ cell lines differentiated into SC and SFC for 14 days. An exceptional SOX2^+^ cell line, GBM B17, differentiated in SC with different serum concentrations, showed a higher percentage of CD105^+^ cells by flow cytometry data. **F** Sorted CD105^+^ cells differentiated in SFC supplemented with b-FGF for 14 days. Differentiated cells were stained with CD105 (red) and SOX2 (green). Bar graphs showing quantification of cell viability of (left lower) and CD105^+^ cell subpopulation (right lower). 5 CD105^+^ cell lines were differentiated in SFC for 14 days. Graph shows CD105^+^ cell lines tolerating SFC but losing CD105 marker positivity. *P < 0.05 **P < 0.01 ***P < 0.001

To isolate and study CD105^+^ cells derived from the GBM preinvasive niche and to clarify under which culturing conditions these cells can be optimally propagated, primary cell cultures were set up using two different protocols: serum condition (SC) and serum-free condition (SFC) (Fig. 2A). SFC cultured cells displayed mainly a spindle-shaped cell morphology and aggregated as spheroids in long-term culture, whereas SC cultured cells initially showed a mixed tripolar or multipolar or flattened morphology and all of them became flatten-enlarged cells in long-term cell culture (Fig. 2B, Additional file 3: Fig S2A). Immunofluorescent (IF) staining identified most of the SFC cultured cells as SOX2^+^Nestin^+^ cells and a few cells showed CD105 positivity. In contrast, SC cultured cells expressed CD105 and Nestin but no SOX2 or SOX9 (Fig. 2B, Additional file 3: Fig S2B). The CD105^+^ cell population was sorted from SC and SFC cultured cell lines by FACS. The percentage of CD105^+^ cells from SC cultured cell lines ranged from 4.4% to 87% (Mean value 37%), and the CD105^+^ cell fraction in SFC cultures varied between 0.13% and 9.4% (Mean 2.8%). Thus, a high proportion of CD105^+^ cells is dependent on SC (*P* < 0.001) in vitro (Fig. 2C).

### GBM-derived CD105^+^ cells share similarities with mesenchymal stem cell-like cells

To phenotypically characterize CD105^+^ cells, we tested cell-type-specific markers recently reported to be expressed on CD105^+^ cells (Additional file 1: Table S2). We found that all low-passage (p3-5) CD105^+^ cells co-expressed Nestin and Vimentin and most of the cells were positive for βIII-tubulin and α-smooth muscle actin (α-SMA) (Fig. 2D). The CD105^+^ cells were negative for macrophage markers: CD163 and CD68; the endothelial cell marker: vWF, microglia markers Iba1 and TMEM119, the fibroblast marker FAP, the mature pericyte marker NG2, the neuronal marker NeuN and the astrocyte marker GFAP, suggesting that GBM CD105^+^ cells might originate from a mesenchymal stem cell (MSC) lineage (Additional file 1: Table S2). To further tie GBM CD105^+^ cells towards a MSC origin, we tested if CD105^+^ cells could differentiate into adipocyte, osteoblast and chondrocyte lineages. After differentiating 8 CD105^+^ cell lines, we observed that all lines could differentiate into osteoblast and 6 of them could generate an adipocyte phenotype, but none of them differentiated into chondrocytes (Additional file 3: Fig S2B, Additional file 1: Table S3). To verify that the CD105^+^Nestin^+^ mesenchymal phenotype exits all along the cell fate predestination, we cultured GBM CD105^+^ cells for one month in one passage and assayed the expression of Ki67, CD105, Nestin and Vimentin. During long-term culture, the morphology of the CD105^+^ cells became flattened and enlarged. At this time point, IF staining was negative for Ki67 but still positive for CD105, Nestin and Vimentin (Additional file 3: Fig S2c). In conclusion, GBM-derived CD105^+^ cells share similarities with mesenchymal stem cell-like cells and they are of distinct lineage from endothelial cells and pericytes.

### Maintenance of CD105^+^ or SOX2^+^ phenotypes depend on different serum culture conditions

Primary culture of CD105^+^ cells or SOX2^+^ cells thus depend on SC and SFC, respectively. To clarify whether serum is essential for preserving the CD105^+^ or SOX2^+^ phenotype, 10^6^ SOX2^+^ cells from 5 primary lines were cultured in 10% FBS media for 14 days. We found that almost all the original SOX2^+^ cells changed into elongated, spindle-shaped, or flatten-enlarged cells. Cell viability assay showed that the growth of 4 SOX2^+^ cell lines ceased while only one cell line kept growing. This latter cell line was placed into SC with different serum concentrations for 14 days. The CD105^+^ cell population derived from this cell line was then quantified by flow cytometry as 25.2% in 2% FBS culture condition, 26.2% in 10% FBS and 31.8% in 20% FBS. Ki67 staining verified that these cells kept proliferating (Additional file 3: Fig S2D). No CD105^+^ cells could be found from the other 4 lines. IF assay demonstrated that all the cells in SC lost the SOX2 phenotype (Fig. 2E). Further, we seeded 10^6^ GBM CD105^+^ cells from 5 primary cell lines into SFC and supplemented with 20 ng/ml b-FGF per day for up to 14 days. CD105^+^ cells detached and developed as sphenoids in SFC. Cell viability assay demonstrated that all CD105^+^ cell lines kept growing in SFC. However, the cells lost their CD105 marker positivity during SFC culture as assayed by flow cytometry. SOX2 expression was absent on CD105^+^ sphenoids by IF examination, indicating that CD105^+^ cells are clearly distinguished from SOX2^+^ GBM cells (Fig. 2F). Thus, in contrast to previously characterized SOX2^+^ GCSs, CD105 cells depend on serum for in vitro expansion.

### GBM CD105^+^ cells have cancer stem-like potency in vitro

**Fig. 3:**
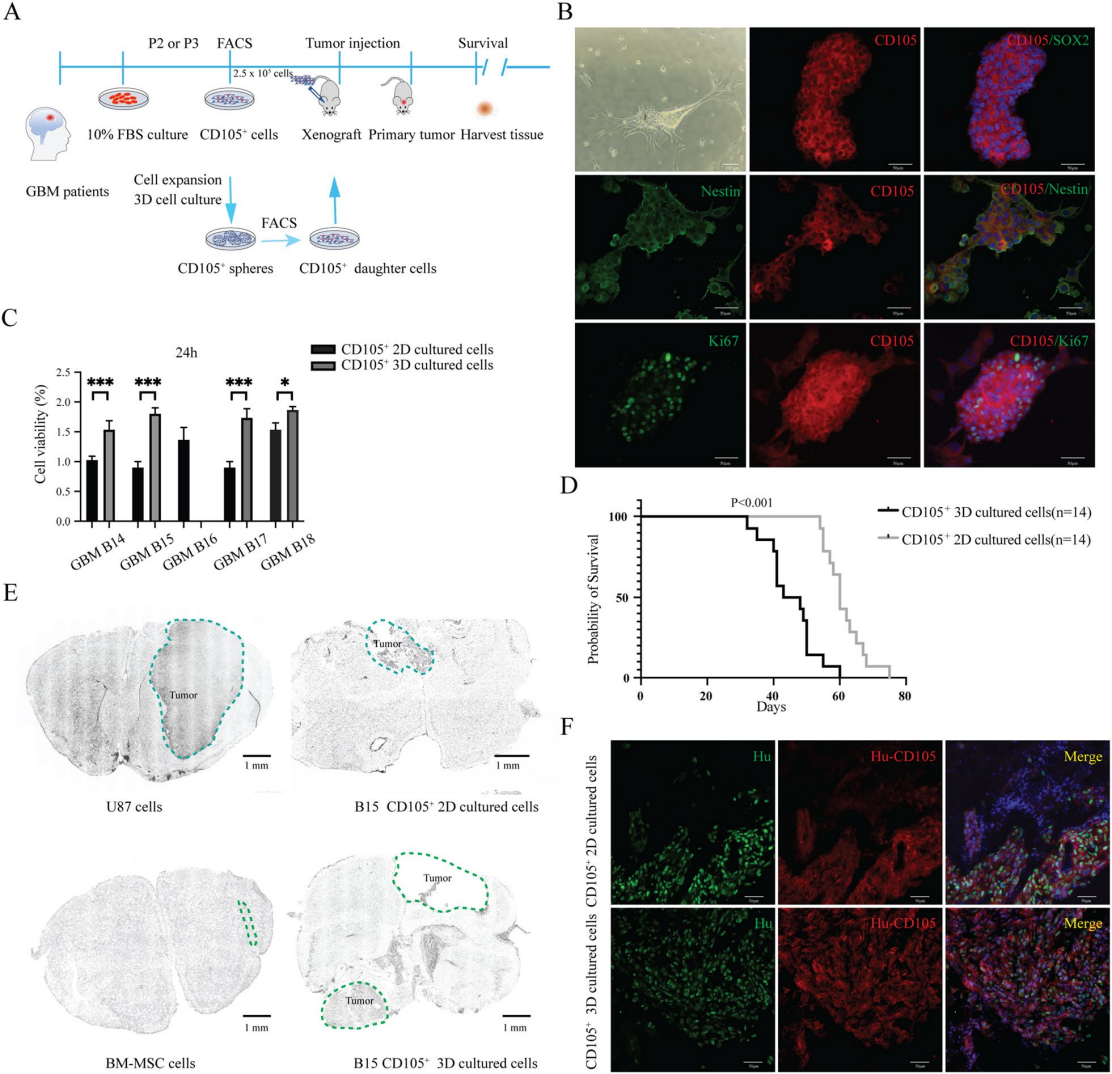
CD105^+^ cell stemness and tumorigenicity assay. **A** Schematic of intracranial transplantation for CD105^+^ cells. Sorted primary CD105^+^ cell lines (n=5) cultured in 2D and 3D conditions. 250,000 cells were injected into NSG mouse brains. Brain tissue was harvested immediately following the loss of each animal. MSCs cell line from a healthy donor and U87 cell line was used as controls (n=5 in each group). **B** Sphere formation assay of GBM CD105^+^ cells. CD105^+^ cells were cultured in 3D conditions for 14 days and tumorspheres captured under the brightfield or fluorescence microscopy. IF double staining shows CD105 double stained together with SOX2, Nestin and Ki67. **C** Comparison of the cell viability of CD105^+^ cells between 2D and 3D cultured conditions. 1000 of 2D or 3D CD105^+^ cells cultured for 24h. Cell viability assay based on PrestoBlue fluorescence intensity of each CD105^+^ cell line. *** *P* < 0.001, *P < 0.05. **D** Kaplan–Meier survival curves comparing 2D or 3D cultured CD105^+^ cells xenografts. **E** Scans of mouse brain tumor sections. **F** Brain sections of xenografted mice transplanted with 2D or 3D cultured CD105^+^ cells stained with CD105 (red) and Hu (green) and DAPI (blue)

To assess the stem cell potential of GBM CD105^+^ cells, we performed in vitro sphere formation assay. We sorted CD105^+^ cell lines (n = 5) and placed them into 3D culture conditions (Fig. 3A). All lines expanded and formed tumor spheres except line GBM B16. IF assay showed that CD105^+^ spheres retained the CD105 and Nestin positivity and SOX2 negative profiles, clearly distinguishing them from SOX2^+^ Nestin^+^ CSC spheres. Ki67 staining proved consistent proliferation of CD105^+^ spheres (Fig. 3B). We next dissociated the CD105^+^ spheres and resorted CD105^+^ cells. Compared with the unsorted primary cancer cells, CD105^+^ sphere cells displayed significantly higher proliferation rates (Fig. 3C). These experiments proved that primary GBM CD105^+^ cells could self-renew and generate robust spheres, indicating a cancer stem-like cell phenotype.

### GBM CD105^+^ cells generate tumors in vivo

To assay the in vivo tumorigenic potential of GBM-derived CD105^+^ cells, the GBM B15 cell CD105^+^ cell line was grafted as 2D cultured cells and 3D cultured cells into NSG mice’s brain (n = 5 per group). We simultaneously used human BM-MSCs, another CD105^+^ cell line from healthy donors, and U87 cells as control cell lines. The mice implanted with CD105^+^ cells and CD105^+^ spheres had significantly shorter survival than the BM-MSCs group, but longer than the U87 group (Additional file 3: Fig S3). Moreover, the CD105^+^ 3D cell culture group died earlier than the CD105^+^ 2D cell culture group (Fig. 3D). Analysis of brain of mice implanted with CD105^+^ cells as well as CD105^+^ spheres all contained tumors (Fig. 3E). Further, we tracked the CD105^+^ cells by human specific antibodies. IF detection demonstrated that the cells in the tumor bulk originated from human cells and displayed a prominent CD105^+^ expression and no SOX2 immunoreactivity (Fig. 3F). Collectively, the in vivo data proved that GBM CD105^+^ cells could self-renew and give rise to tumor bulk.

### GBM CD105^+^ cells display a mutational landscape characteristic of tumors

Whole exome sequencing was performed in 5 GBM patient derived CD105^+^ cell lines to identify tumor associated mutations and to assess the degree of exome homology compared to GBM. The quality of the sequencing is summarized in Additional file 1: Tables S4, S5 and Additional file 3: Fig S4A-F. On average, we obtained 291 million rough aligned reads for each cell line and the coverage was 379.3x. An average of 387.6 million input reads were mapped to the human genome NCBI36/hg 39 reference assembly. The average read length was 355 bases (Additional file 2: Table S6).

**Fig. 4:**
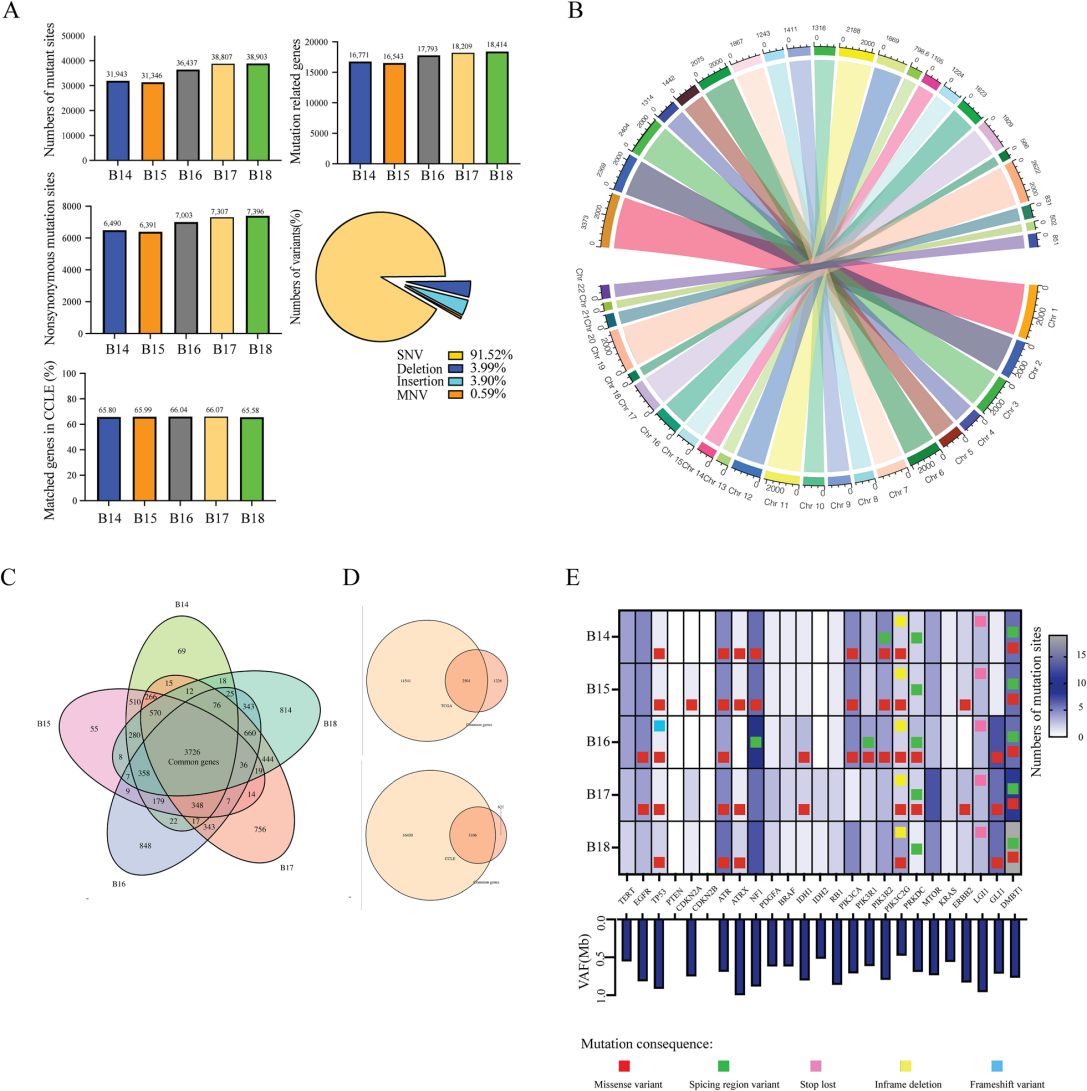
Exome sequencing of GBM CD105^+^ cells. **A** The number of mutations, annotated genes and the rate of mutant genes matching into the CCLE cancer database were analyzed from the exome sequencing data of 5 GBM CD105^+^ cell lines. Pie chart quantifying the subtype of mutations. **B** Circos chart showing the distribution of mutant genes in each chromosome. The area represents the number of mutant genes. **C** Veen chart displaying common mutant genes among 5 GBM CD105^+^ cell lines. **D** Mutant genes matching TCGA and CCLE databases.** E** The mutant genes of GBM CD105^+^ cells matching with GBM hallmark genes from COSMIC. Upper panel: Representation of mutation types. Lower panel: Subclone variant allele frequencies (VAF)

We further analyzed the data by the Illumina DRAGEN platform. 35,487 variants were detected per cell line: 92% of them were single-nucleotide variants (SNV), 4.0% were deletions, 3.9% were insertions and 0.59% were multiple-nucleotide variants (MNV). On average of 17,546 genes associated with genetic mutation sites. Among them, 7007 were identified as genes with nonsynonymous mutation sites. We compared the annotated genes of our data with the Cancer Cell Line Encyclopedia (CCLE; https://sites.broadinstitute.org/ccle) which included 1750 cell lines from 37 different cancer cell lineages. On average, 66% genes of our data matched the genes in CCLE (Fig. 4A). Chromosome 1 and 19 contained the largest number of mutant genes in GBM CD105^+^ cell lines (Fig. 4B), however, the number of mutant genes distributed differently between each cell line and chromosome. To explore common genome characteristics among the 5 different CD105^+^ cell lines, we compared nonsynonymous mutations of each cell line and annotated 3712 shared genes based on shared mutations (Fig. 4C, Additional file 2: Table S6). We matched these genes in GBM TCGA and CCLE databases. On average of 67% of them matched into TCGA and 83% matched CCLE, respectively. These data suggest that GBM pre-invasive niche derived CD105^+^ cells are highly homologous to cancer cell lines (Fig. 4D).

To further demonstrate the relation between CD105^+^ cell lines and GBM, we matched the genes which are the most common mutations in GBM patients from the Catalogue of Somatic Mutations in Cancer (COSMIC) [26] database with our data. Mutations in TP53, ATR, PIK3C2G, PRKDC and DMBT1 were present in all the GBM CD105^+^ cell lines while PTEN and CDKN2B were negative. These results suggest that GBM CD105^+^ cells are transformed and potentially tumorigenic.

Variant frequencies among the observed genes were analyzed and all values were over 50%, indicating that these mutations were potential germline mutations and may be passed onto its subclones (Fig. 4E). Mutant genes were correlated to the clinical prognosis. We listed the 20 genes with the highest number of SNVs and linked these to the patient survival data from GBM TCGA (Additional file 2: Table S6). We did not find any direct link between the gene mutation and patient overall survival (Additional file 3: Fig S3G). In brief, exome sequencing indicated that GBM CD105^+^ cells display mutations indicative of a cancer genotype.

### GBM CD105^+^ cells influence the TME

**Fig. 5:**
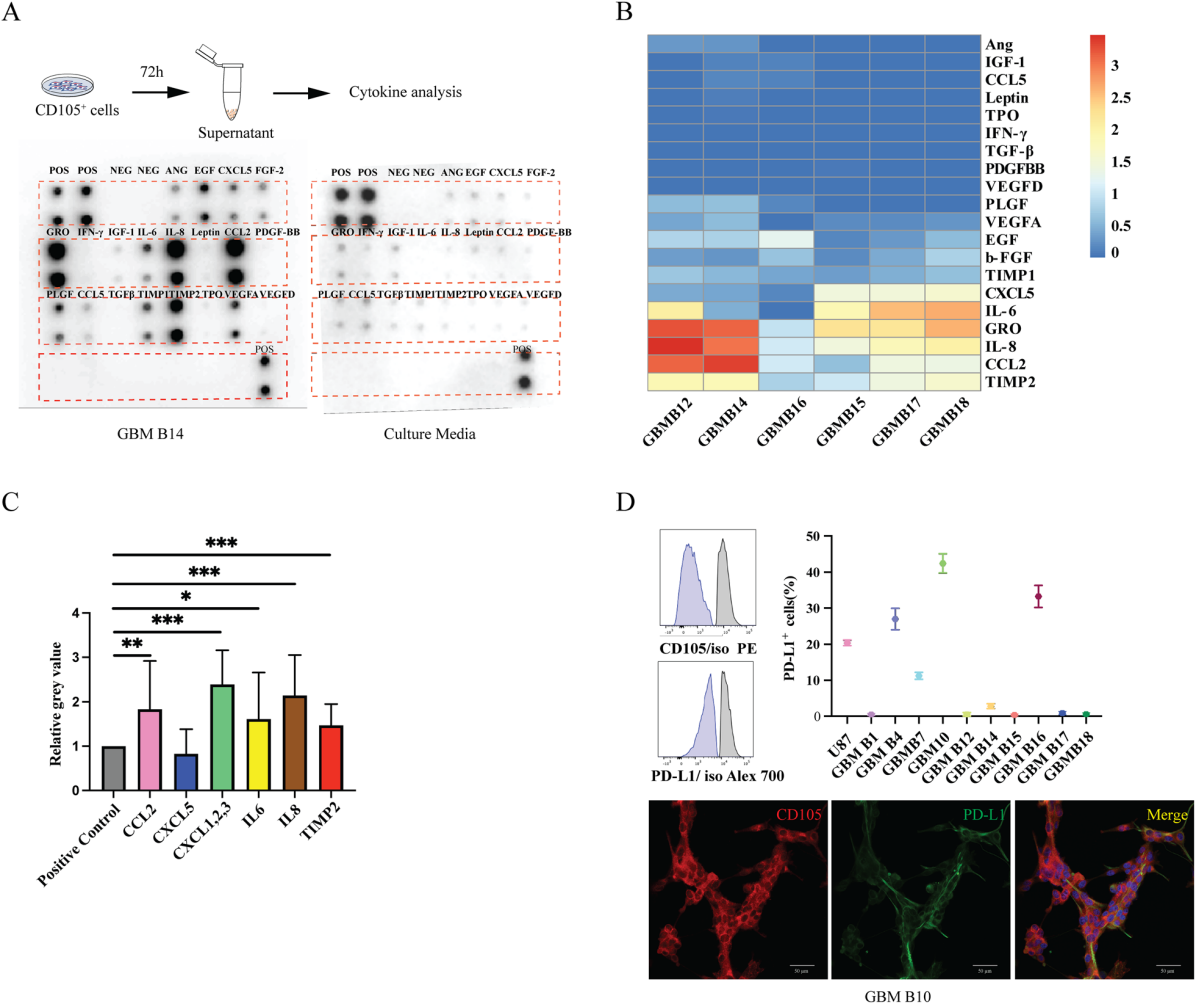
In vitro functional assays of the crosstalk between CD105^+^ cells and TME. **A** Angiogenic and immunosuppressive factors detected by antibody assay. *POS* positive control, *NEG* negative control, *ANG* angiogenin, *EGF* epidermal growth factor, *CXCL5 C-X-C* Motif Chemokine Ligand 5, *FGF-2* fibroblast growth factor-2, *GRO* growth-related oncogene, *IFN-γ* interferon gamma, *IGF* insulin-like growth factor, *IL-6* interleukin 6, *IL-8* interleukin 8, *CCL* C-C Motif Chemokine Ligand, *PDGF-BB* platelet-derived growth factor B-chain homodimer, *PLGF* placenta growth factor, *TGFβ* transforming growth factor-β, *TIMP* tissue inhibitor of metalloproteinase, *TPO* thyroid peroxidase, *VEGF* vascular endothelial growth factor. **B** Heatmap of 20 angiogenic and immunosuppressive factors identified on 6 GBM CD105^+^ cell lines. Protein expressions are displayed as colors ranging from red to blue as shown in the key. **C** Quantification of the overexpressed proteins on CD105^+^ cells (n = 6 cell lines). *P < 0.05, **P < 0.01, ***P < 0.001. **D** PD-L1 expression assays on GBM CD105^+^ cells. Flow cytometry data showing the differential PD-L1 expression on CD105^+^ cell lines. Lower panels: IF staining verifying the coexpression of CD105 (red) and PD-L1 (green)

To explore potential crosstalk between GBM CD105^+^ cells and the TME, angiogenesis and immune cytokine assays were performed on the supernatant collected from GBM CD105^+^ cell culture medium (n = 6) after 72 h culture. (Fig. 5A) Compared with control (culture medium only), immune cytokines such as IL-8, CCL2, GRO, TIMP2 were found at strong immunopositivity in the supernatant. More modest levels of IL-6 and CXCL5 were found, while most angiogenesis-related factors were absent (Fig. 5B). Semi-quantifying the grey value on ELISA blots representing each cytokine present in the supernatant, we confirmed high IL-6, IL-8, CCL2, GRO and TIMP2 protein levels, indicating a possible function in recruiting immune suppressive cells but not in angiogenesis (Fig. 5C). Further, we interrogated the expression of programmed death-ligand 1 (PD-L1), an immune-modulating agent, on GBM CD105^+^ cell lines (n = 10) and the U87 cell line by both IF and flow cytometry assays. The PD-L1 protein showed variable expression in each GBM CD105^+^ cell line, ranging from 0.3% to 42.4% positive cells out of the total cells (Fig. 5D). In summary, cytokine assays showed that several powerful immunosuppressive and protumorigenic cytokines are produced by GBM CD105^+^ cells.

### Drug screening identified potential chemotherapeutics against GBM CD105^+^ cells

**Fig. 6:**
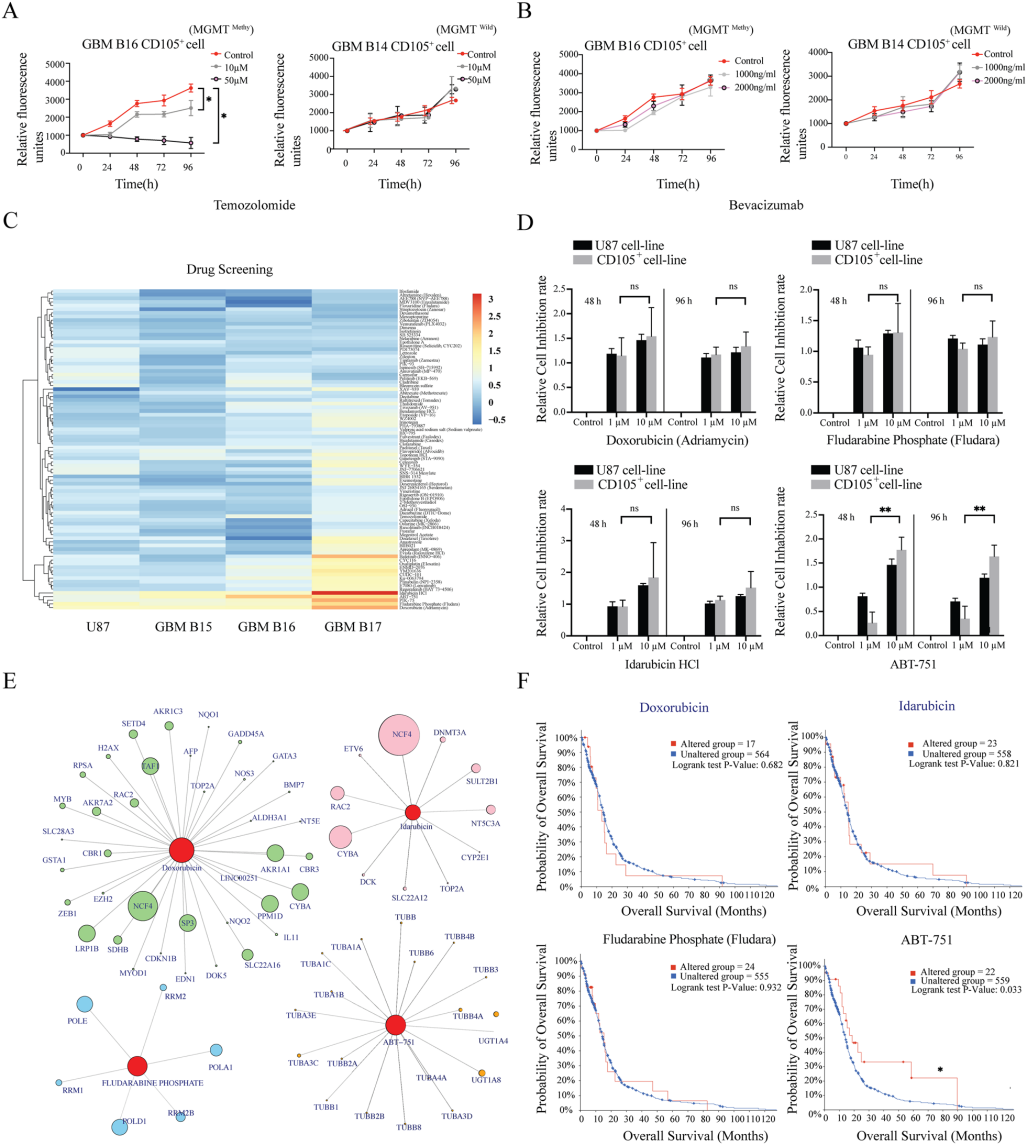
Drug screening in vitro. **A**, **B** Cell toxicity assays on CD105^+^ cells lines. Different concentrations of temozolomide (**A**) and bevacizumab (**B**) on MGMT promoter methylated CD105^+^ cell line (GBM B16) and MGMT wildtype CD105^+^ cell line (GBM B14) cultured for 96h. Fluorescence units represent the cell viability detected at 24 h, 48 h, 72 h and 96 h. Control cells were kept in culture medium without adding any drug. **C** Heatmap showing the toxicity of 88 clinical compounds (10μM) against 3 GBM CD105^+^ cell lines and U87 cell line. Cell toxicities are displayed by the range of the colors from red to blue as high to low. The rows are clustered using correlation distance. **D** Cell toxicity assay showing the effect of different concentrations of Doxorubicin, Idarubicin HCl, Fludara and ABT-751 on CD105^+^ and U87 cells. Cell viability was detected at 48 h and 96 h. **P < 0.01. **E** Interaction plot between drugs and genes. Each circle represents a gene, and its area symbolizes relevance.** F** Kaplan–Meier curves showing the relation between patient survival and drug-interacted genes as analyzed using patient data from TCGA database. *P* value is calculated by logrank test. *P < 0.05

Temozolomide (TMZ) is the most frequently used chemotherapeutic agent in GBM patients, and accordingly, we analyzed the GBM CD105^+^ cell response to TMZ. Because the TMZ effect is highly depending on the methylation status of the DNA repair enzyme O(6)-methylguanine-DNA methyltransferase (MGMT) promoter, we selected two different GBM CD105^+^ cell lines, one cell line derived from a patient diagnosed with methylated MGMT promoter and another cell line without methylated MGMT promoter (wildtype). We incubated GBM CD105^+^ cells with different drug concentrations, detecting the cell viability every 24 h for 96 h. GBM CD105^+^ cell line with MGMT promoter methylation showed a significant dose-dependent reduction in proliferation in response to TMZ. In contrast, the wildtype persisted proliferating (*P* < 0.05) (Fig. 6A, Additional file 3: Fig S6A). To further test TMZ toxicity on another GSC subpopulation, we compared TMZ on SOX2^+^ cell lines from the GBM patients with wildtype or methylated MGMT promoter. TMZ also proved effective on the SOX2^+^ cell line with methylated MGMT promoter whereas SOX2^+^MGMT wildtype GSC s were resistant. Interestingly, in contrast to tumor endothelium, the CD105^+^ cells were resistant to the VEGF-A inhibitor Bevacizumab, underscoring that CD105^+^ stem-like cells are not identical to GBM endothelial cells (Fig. 6B, Additional file 3: Fig S6B). SOX2^+^ cells lines, irrespective of methylation status, also showed resistance to bevacizumab (Additional file 3: Fig S6C).

To identify effective pharmaceuticals specifically against GBM CD105^+^ cells, we next performed a drug screening with 88 clinical drugs selected for their structural diversity. Three GBM CD105^+^ cell lines and the muti-drug resistant U87 cell line was used. The response was assessed by detecting cell viability (Prestoblue) after 48 h and 96 h exposure to 1 µM and 10 µM drug. Drug sensitivity was calculated as cell viability ratio between cells exposed to drug compared to cells in culture medium only. We identified Doxorubicin, Idarubicin, Fludarabine and ABT-751 to exhibit a robust toxic effect on GBM CD105^+^ cells (Fig. 6C). We further examined the dose-dependent effect of these candidate drugs. 10 µM ABT-751 had a significantly stronger toxic effect on GBM CD105^+^ cells compared to 1 µM ABT-751. Toxic effects of Doxorubicin, Idarubicin, Fludarabine on GBM CD105^+^ cells did not show any dose dependency (Fig. 6D).

In order to explore if ABT-751, Doxorubicin, Idarubicin, Fludarabine have the potential to change expression of key genes in GBM, we predicted drug-gene interactions using the online DGIdb platform. NCF4, CYBA, POLD1, POLD and UGT1A8 were indicated as the genes whose expression were most likely to be affected by the drug candidates (Fig. 6E). Next, we selected each of the 5 genes with the highest interaction score with the drug candidates and assessed the relationship between gene expression and clinical survival. Patients with gene expression affected by ABT-751 had significantly prolonged overall survival compared to those without gene expression alterations, indicating ABT-751 at priority for use in GBM (Fig. 6F). These data provide novel information for selecting effective drug therapy in GBM patients and for delaying tumor recurrence.

## Discussion

GSCs represent a subpopulation of malignant cells exhibiting capabilities of driving tumor progression, self-renewal, differentiation and resisting conventional chemotherapy [27, 28]. In this study, we isolated CD105^+^ cells from the pre-invasive front of human GBM tumors and demonstrate that these cells act as a subpopulation of GBM stem-like cells in vitro and in vivo. Furthermore, we predicted their sensitivity to established chemotherapeutics, presenting potential targeting strategies.

Abnormal vasculature is a crucial feature of GBM [29, 30]. GBM vessels create a tumor microenvironment that, in an intra- and intertumoral heterogeneous manner, include severe hypoxia, acidosis, necrosis and high interstitial pressure and promote tumor progress [31]. CD105 expression is identified as a diagnostic hallmark of GBM vascular structures [32, 33], and a higher expression level of CD105 correlates with a shorter overall survival time [34]. Our own result proved that CD105^+^Nestin^+^ cells exist outside of the GBM margin and might be related with tumor recurrence.

Maintenance and enrichment of primary GBM CSCs isolated from operative GBM specimens need culture under serum-free, stem cell conditions [35]. Although no universally accurate markers for GBM stem cells exist, among the most frequently used markers for detection of GBM cancer stem-like cells, are SOX2 and Nestin [36–38]. We set up GBM primary cell lines under SFC and purified a CD105^+^ subpopulation from these cells. Most unsorted, primary cells were SOX2^+^Nestin^+^ and by flow cytometry we found only 2.8% CD105^+^ cells. In contrast, when GBM primary cell lines were cultured in 10% FBS condition, we found 37% CD105^+^ cells and only a minor fraction of SOX2^+^ cells. Because of the controversy of a lineage relationship between neural stem cells and endothelial cells, we further tested cell differentiation capabilities in SC and SFC. We found that SOX2^+^Nestin^+^ cell lines lost SOX2^+^ expression upon differentiation in SC and most of them could not differentiate into CD105^+^ cells. Only one exceptional patient case showed 26.2% CD105^+^ cells following differentiation in SC, but the original cell line also contained a high percentage (9.4%) of CD105^+^ cells, suggesting the CD105^+^ cells of this exceptional case are subclones of the original CD105^+^ cells and not due to differentiation of SOX2^+^ cells. We also found that CD105^+^ cells lost CD105 expression when cultured in SFC. We further characterized the markers of CD105^+^ cells, and all CD105^+^ cells co-expressed Nestin and Vimentin, most of the cells were positive for βIII-tubulin and α-SMA, and all of them were negative for NG2, FAP and SOX2. All these data suggest CD105^+^ cells are not derived from tumor endothelium, pericytes, fibroblasts or of neural stem cell lineage, and clearly distinguish these cells from previous SFC cultured CSCs [27, 39, 40].

Recently, stemness identification has predominantly relied on xenotransplantation assays using patient-derived cell lines and subsequent sorting for the presence or absence of candidate markers after tumor bulk formation [35, 41]. A complementary, non-marker method to detect cell stemness is to test tumor spheroid formation in vitro following single-cell plating [42–44]. Our experiments demonstrate GBM CD105^+^ cells from tumor spheroids in vitro. As for differentiation, all GBM CD105^+^ cell lines can differentiate into osteocytes and a subset of them can differentiate into adipocytes. The present study presents proof that GBM CD105^+^ cells give rise to a tumor bulk in an orthotopic xenograft model and that virtually all the tumor cells maintain the CD105 expression in vivo. Together, these results provide robust proof that GBM CD105^+^ cells have stemness potency and that they could be referred to as a tumor stem-like cell subpopulation. However, CD105^+^ cells in our hands differentiate into cells with unclear tumorigenic potential in long-term cell culture, so the methods to maintain long-term stemness in vitro is still unclear.

Previous genomic studies have accumulated GBM specific aberrations in somatic mutations, gene expression, and epigenetic alternations collected within TCGA, COSMIC and CCLE databases [45–47]. The whole-exome mutation pattern of GBM CD105^+^ cell lines match 83% of genes in the CCLE registry, indicating a high homology between GBM CD105^+^ cells and cancer cells [45]. We selected the 25 most common mutations occurring in GBM samples from COSMIC and compared these mutations with the CD105^+^ cell lines. TP53, ATR, PIK3C2G, PRKDC and DMBT1 genes, identified as frequently mutated in GBM by other studies [48], displayed mutations in CD105^+^ cell samples. The TP53 gene, encoding the tumor suppressor P53 protein, acts as guardian of genome, and mutations are a found in many types of tumor cells where it acts to drive cancerous transformation [49]. ATR protein is a key kinase in the DNA damage response and responsible for sensing DNA replications stress. Evidence indicates that ATR mutations occur in numerous types of tumors [50]. Mutations of PIK3C2G, PRKDC and DMBT1 are also commonly found in cancer patients [51, 52].

CD105 was investigated as an antiangiogenic therapeutic target for several years. The antibody-based drug TRC105 preclinically inhibits tumor growth but was unsuccessful in clinical trials [17]. In line with this, the present study proved that GBM cell lines could keep proliferation without CD105 expression. We tested 88 clinical compounds for their effects on CD105^+^ cells and found that ABT-751 was effective for both CD105 + cell lines and the U87 cell line. ABT 751, a bioavailable tubulin-binding agent, has already shown an antitumor effect in recent preclinical studies [53]. However, previous clinical studies revealed limited CSF penetration of ABT751 [54]. Possibly, local intratumoral delivery of ABT-751 via implanted catheters or a drug-releasing biocompatible matrix could solve the problem of poor blood–brain barrier permeability.

**Fig. 7:**
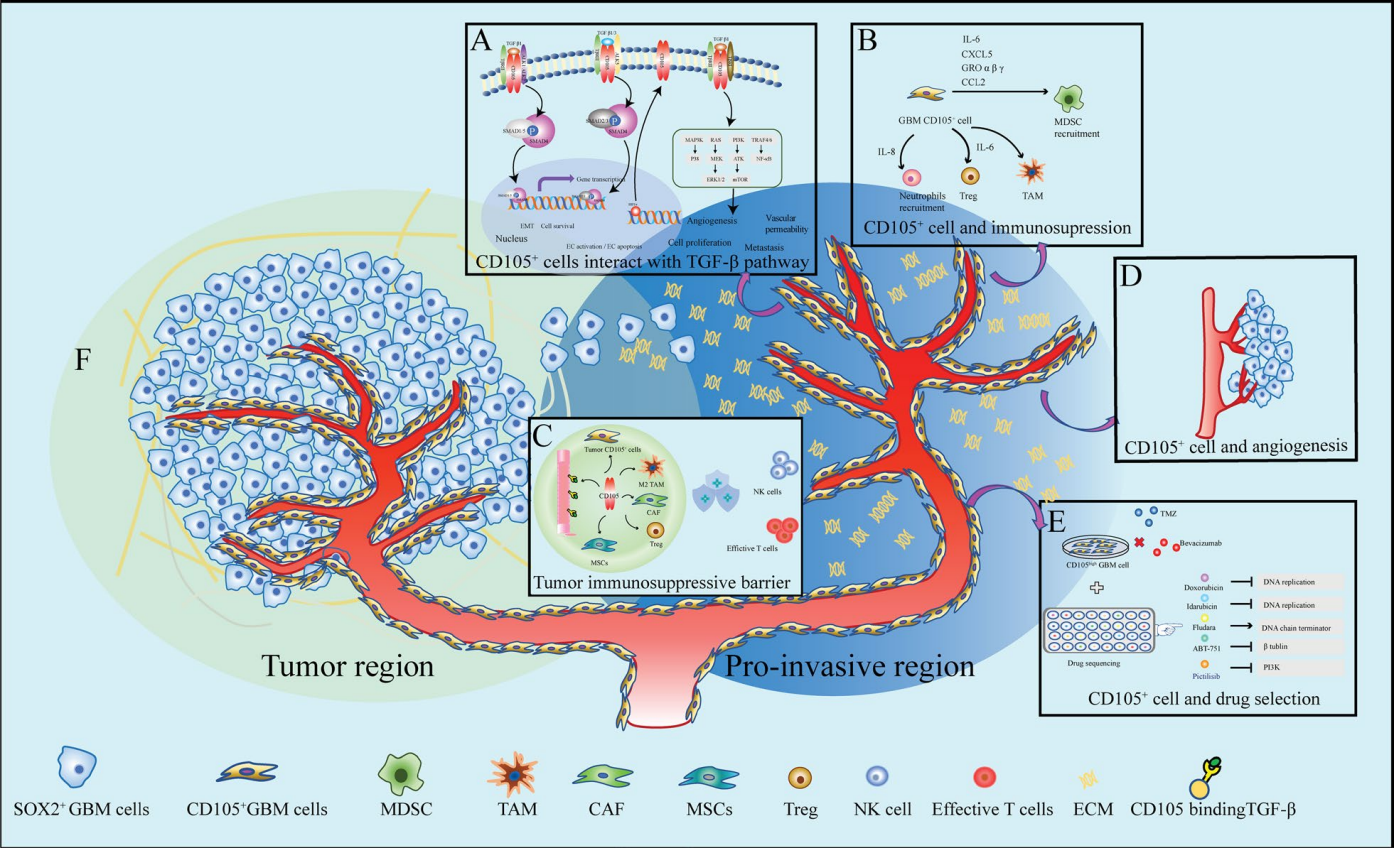
Schematic illustrating multiple roles of CD105^+^ cells in GBM. **A** CD105^+^ cell interacts with the TGF-β pathway. CD105, a receptor of TGF-β, can affect angiogenesis, vascular permeability and cell proliferation by binding different TGF-β subtypes. **B**, **C** CD105^+^ cell shapes the tumor immunosuppressive microenvironment. Cytokines secreted by GBM CD105^+^ cells share the function of recruiting immunosuppressive cells. CD105^+^ cells impair immune cell function and contribute to creating an immunosuppressive barrier. **D** CD105 constitute a marker of GBM vasculature. **E** In vitro drug sensitivity screening of CD105^+^ cells. CD105^+^ cells are resistant to conventional drugs: TMZ and AVZ but sensitive to ABT-751, Doxorubicin, Idarubicin and Fludarabine. **F** Schematic depicts the “Seed and soil” rational for GBM growth. Based on the distribution and function of CD105^+^ cells, we predict CD105^+^ cells may release extracellular matrix (ECM), initiating a tumor tolerant microenvironment and thereby promoting tumor progression

The mechanisms of GBM initiation and expansion are still unclear. Tumor initiation may result from normal neural stem cells accumulating oncogenic mutations that transform these cells into cancer stem-like cells capable of generating cancer cell subclones [54–56]. An alternative theory, based on Paget's "seed and soil" hypothesis and demonstrated by many studies, states that tumor initiation and expansion are held silent until a permissive environment is generated [57–59]. Therefore, we incline to believe that the TME can trigger carcinogenesis as a result of conversion from normal stem cell to CSCs. Recent studies demonstrated that extracellular matrix (ECM) from CSCs act as a precursor for the early stage of tumorigenesis and that the ECM triggers normal to tumoral microenvironment transition [60, 61]. Questions regarding which subpopulation of CSCs secreting pro-tumoral ECM that may initiate the process of tumorigenesis should further be clarified. In this study, we found that there exist numerous CD105^+^ cells outside of the tumor border but very few SOX2^+^ Nestin^+^ cells which may be considered as the main tumor cell component in patient peritumoral samples. This may provide a clue to find out a relatively quiescent subpopulation of tumor (stem) cells, remaining outside of the tumor border within the pre-invasive niche, are responsible for GBM recurrence. Interestingly, we detected high expression of IL-6, IL-8, CCL2, GRO and TIMP2 in CD105^+^ cell culture media and the function of all these proteins point to the recruitment of immunosuppressive cells [62]. Thus, GBM CD105^+^ cells may change the local microenvironment to a tumor tolerating and permissive TME, paving the way for tumor regrowth (Fig. 7). However, the possible mechanisms influencing the TME exerted by CD105^+^ cells need further study.

## Supplementary Information

**Additional file 1:** **Table S1**. Clinical Information regarding GBM cell lines. **Table S2**. Cell markers used for GBM CD105^+^ cell characterization. **Table S3**. Differentiation assay of GBM CD105^+^ cells. **Table S4**. Panel metrics summary of GBM CD105^+^ cell exome sequencing. **Table S5**. Variant calling metrics of GBM CD105^+^ cell exome sequencing.

**Additional file 2.** Supplementary **Table S6**.

**Additional file 3.** Supplementary Figures: **Fig. S1, S2, S3, S4, S6**.

## References

[1] Bexell D, Gunnarsson S, Nordquist J, Bengzon J (2007). Characterization of the subventricular zone neurogenic response to rat malignant brain tumors. Neuroscience.

[2] Krex D, Klink B, Hartmann C, Von Deimling A, Pietsch T, Simon M (2007). Long-term survival with glioblastoma multiforme. Brain.

[3] Ramirez YP, Weatherbee JL, Wheelhouse RT, Ross AH (2013). Glioblastoma multiforme therapy and mechanisms of resistance. Pharmaceuticals.

[4] Kondo T, Setoguchi T, Taga T (2004). Persistence of a small subpopulation of cancer stem-like cells in the C6 glioma cell line. Proc Natl Acad Sci.

[5] Wang R, Chadalavada K, Wilshire J, Kowalik U, Hovinga KE, Geber A (2010). Glioblastoma stem-like cells give rise to tumour endothelium. Nature.

[6] Suvà ML, Rheinbay E, Gillespie SM, Patel AP, Wakimoto H, Rabkin SD (2014). Reconstructing and reprogramming the tumor-propagating potential of glioblastoma stem-like cells. Cell.

[7] Jackson M, Hassiotou F, Nowak A (2015). Glioblastoma stem-like cells: at the root of tumor recurrence and a therapeutic target. Carcinogenesis.

[8] Pallini R, Ricci-Vitiani L, Banna GL, Signore M, Lombardi D, Todaro M (2008). Cancer stem cell analysis and clinical outcome in patients with glioblastoma multiforme. Clin Cancer Res.

[9] Vanderbeek AM, Rahman R, Fell G, Ventz S, Chen T, Redd R (2018). The clinical trials landscape for glioblastoma: Is it adequate to develop new treatments?. Neuro Oncol.

[10] Cheifetz S, Bellón T, Calés C, Vera S, Bernabeu C, Massague J (1992). Endoglin is a component of the transforming growth factor-beta receptor system in human endothelial cells. J Biol Chem.

[11] Li DY, Sorensen LK, Brooke BS, Urness LD, Davis EC, Taylor DG (1999). Defective angiogenesis in mice lacking endoglin. Science.

[12] Smith SJ, Tilly H, Ward JH, Macarthur DC, Lowe J, Coyle B (2012). CD105 (Endoglin) exerts prognostic effects via its role in the microvascular niche of paediatric high grade glioma. Acta Neuropathol.

[13] Paauwe M, Heijkants RC, Oudt CH, Van Pelt GW, Cui C, Theuer C (2016). Endoglin targeting inhibits tumor angiogenesis and metastatic spread in breast cancer. Oncogene.

[14] Hossain A, Gumin J, Gao F, Figueroa J, Shinojima N, Takezaki T (2015). Mesenchymal stem cells isolated from human gliomas increase proliferation and maintain stemness of glioma stem cells through the IL-6/gp130/STAT3 pathway. Stem cells.

[15] Grange C, Tapparo M, Collino F, Vitillo L, Damasco C, Deregibus MC (2011). Microvesicles released from human renal cancer stem cells stimulate angiogenesis and formation of lung premetastatic niche. Can Res.

[16] Plate KH, Scholz A, Dumont DJ (2012). Tumor angiogenesis and anti-angiogenic therapy in malignant gliomas revisited. Acta Neuropathol.

[17] Liu Y, Paauwe M, Nixon AB, Hawinkels LJ (2020). Endoglin targeting: lessons learned and questions that remain. Int J Mol Sci.

[18] Blanchet L, Krooshof PWT, Postma GJ, Idema AJ, Goraj B, Heerschap A (2011). Discrimination between metastasis and glioblastoma multiforme based on morphometric analysis of MR images. Am J Neuroradiol.

[19] Petrecca K, Guiot MC, Panet-Raymond V, Souhami L (2013). Failure pattern following complete resection plus radiotherapy and temozolomide is at the resection margin in patients with glioblastoma. J Neurooncol.

[20] De Bonis P, Anile C, Pompucci A, Fiorentino A, Balducci M, Chiesa S (2013). The influence of surgery on recurrence pattern of glioblastoma. Clin Neurol Neurosurg.

[21] Ledri M, Sørensen AT, Madsen MG, Christiansen SH, Ledri LN, Cifra A (2015). Differential effect of neuropeptides on excitatory synaptic transmission in human epileptic hippocampus. J Neurosci.

[22] Svensson A, Ramos-Moreno T, Eberstål S, Scheding S, Bengzon J (2017). Identification of two distinct mesenchymal stromal cell populations in human malignant glioma. J Neurooncol.

[23] Andrews, S (2010) FastQC: a quality control tool for high throughput sequence data.

[24] Schneider CA, Rasband WS, Eliceiri KW (2012). NIH Image to ImageJ: 25 years of image analysis. Nat Methods.

[25] Freshour SL, Kiwala S, Cotto KC, Coffman AC, McMichael JF, Song JJ (2021). Integration of the drug-gene interaction database (DGIdb 40) with open crowdsource efforts. Nucleic Acids Res.

[26] Tate JG, Bamford S, Jubb HC, Sondka Z, Beare DM, Bindal N (2019). COSMIC: the catalogue of somatic mutations in cancer. Nucleic Acids Res.

[27] Reya T, Morrison SJ, Clarke MF, Weissman IL (2001). Stem cells, cancer, and cancer stem cells. Nature.

[28] Stieber D, Golebiewska A, Evers L, Lenkiewicz E, Brons NH, Nicot N (2014). Glioblastomas are composed of genetically divergent clones with distinct tumourigenic potential and variable stem cell-associated phenotypes. Acta Neuropathol.

[29] Brandenburg S, Müller A, Turkowski K, Radev YT, Rot S, Schmidt C (2016). Resident microglia rather than peripheral macrophages promote vascularization in brain tumors and are source of alternative pro-angiogenic factors. Acta Neuropathol.

[30] Cheng L, Huang Z, Zhou W, Wu Q, Donnola S, Liu JK (2013). Glioblastoma stem cells generate vascular pericytes to support vessel function and tumor growth. Cell.

[31] Wolf KJ, Chen J, Coombes JD, Aghi MK, Kumar S (2019). Dissecting and rebuilding the glioblastoma microenvironment with engineered materials. Nat Rev Mater.

[32] Kong X, Wang Y, Liu S, Xing B, Yang Y, Li Y (2016). CD105 over-expression is associated with higher WHO grades for gliomas. Mol Neurobiol.

[33] Mikkelsen VE, Solheim O, Salvesen Ø, Torp SH (2021). The histological representativeness of glioblastoma tissue samples. Acta Neurochir.

[34] McGahan BG, Neilsen BK, Kelly DL, McComb RD, Kazmi SA, White ML (2017). Assessment of vascularity in glioblastoma and its implications on patient outcomes. J Neurooncol.

[35] Singh SK, Hawkins C, Clarke ID, Squire JA, Bayani J, Hide T (2004). Identification of human brain tumour initiating cells. Nature.

[36] Basu-Roy U, Bayin NS, Rattanakorn K, Han E, Placantonakis DG, Mansukhani A (2015). Sox2 antagonizes the Hippo pathway to maintain stemness in cancer cells. Nat Commun.

[37] Mamun MA, Mannoor K, Cao J, Qadri F, Song X (2020). SOX2 in cancer stemness: tumor malignancy and therapeutic potentials. J Mol Cell Biol.

[38] Lenting K, Verhaak R, Ter Laan M, Wesseling P, Leenders W (2017). Glioma: experimental models and reality. Acta Neuropathol.

[39] You WK, Yotsumoto F, Sakimura K, Adams RH, Stallcup WB (2014). NG2 proteoglycan promotes tumor vascularization via integrin-dependent effects on pericyte function. Angiogenesis.

[40] Higashino N, Koma YI, Hosono M, Takase N, Okamoto M, Kodaira H (2019). Fibroblast activation protein-positive fibroblasts promote tumor progression through secretion of CCL2 and interleukin-6 in esophageal squamous cell carcinoma. Lab Invest.

[41] Scott CE, Wynn SL, Sesay A, Cruz C, Cheung M, Gaviro MVG (2010). SOX9 induces and maintains neural stem cells. Nat Neurosci.

[42] Schatton T, Frank NY, Frank MH (2009). Identification and targeting of cancer stem cells. BioEssays.

[43] Shaheen S, Ahmed M, Lorenzi F, Nateri AS (2016). Spheroid-formation (colonosphere) assay for in vitro assessment and expansion of stem cells in colon cancer. Stem Cell Rev Rep.

[44] Ishiguro T, Ohata H, Sato A, Yamawaki K, Enomoto T, Okamoto K (2017). Tumor-derived spheroids: relevance to cancer stem cells and clinical applications. Cancer Sci.

[45] Masica DL, Karchin R (2011). Correlation of somatic mutation and expression identifies genes important in human glioblastoma progression and survival. Can Res.

[46] Ellezam B, Theeler BJ, Luthra R, Adesina AM, Aldape KD, Gilbert MR (2012). Recurrent PIK3CA mutations in rosette-forming glioneuronal tumor. Acta Neuropathol.

[47] Barretina J, Caponigro G, Stransky N, Venkatesan K, Margolin AA, Kim S (2012). The Cancer Cell Line Encyclopedia enables predictive modelling of anticancer drug sensitivity. Nature.

[48] Aldape K, Zadeh G, Mansouri S, von Reifenberger G, Deimling A (2015). Glioblastoma: pathology, molecular mechanisms and markers. Acta Neuropathol.

[49] Robles AI, Harris CC (2010). Clinical outcomes and correlates of TP53 mutations and cancer. Cold Spring Harb Perspect Biol.

[50] Lecona E, Fernandez-Capetillo O (2018). Targeting ATR in cancer. Nat Rev Cancer.

[51] Martincorena I, Campbell PJ (2015). Somatic mutation in cancer and normal cells. Science.

[52] Georgescu MM, Islam MZ, Li Y, Traylor J, Nanda A (2021). Novel targetable FGFR2 and FGFR3 alterations in glioblastoma associate with aggressive phenotype and distinct gene expression programs. Acta Neuropathol Commun.

[53] Morton CL, Favours EG, Mercer KS, Boltz CR, Crumpton JC, Tucker C (2007). Evaluation of ABT-751 against childhood cancer models in vivo. Invest New Drugs.

[54] Cho SY, Fox E, McCully C, Bauch J, Marsh K, Balis FM (2007). Plasma and cerebrospinal fluid pharmacokinetics of intravenously administered ABT-751 in non-human primates. Cancer Chemother Pharmacol.

[55] Vaux DL (2011). In defense of the somatic mutation theory of cancer. BioEssays.

[56] Gupta PB, Chaffer CL, Weinberg RA (2009). Cancer stem cells: Mirage or reality?. Nat Med.

[57] Paget S (1889). The distribution of secondary growths in cancer of the breast. The Lancet.

[58] Fidler IJ (2003). The pathogenesis of cancer metastasis: the seed and soil hypothesis revisited. Nat Rev Cancer.

[59] Langley RR, Fidler IJ (2011). The seed and soil hypothesis revisited—the role of tumor-stroma interactions in metastasis to different organs. Int J Cancer.

[60] Brassart-Pasco S, Brézillon S, Brassart B, Ramont L, Oudart JB, Monboisse JC (2020). Tumor microenvironment: extracellular matrix alterations influence tumor progression. Front Oncol.

[61] Henke E, Nandigama R, Ergün S (2020). Extracellular matrix in the tumor microenvironment and its impact on cancer therapy. Front Mol Biosci.

[62] Lin WW, Karin M (2007). A cytokine-mediated link between innate immunity, inflammation, and cancer. J Clin Investig.

