# Supplementary material for: Glioblastoma CD105+ cells define a SOX2− cancer stem cell-like subpopulation in the pre-invasive niche
Source: Acta Neuropathol Commun. 2022 Aug 29;10:126. doi: 10.1186/s40478-022-01422-8 (PMC9426031; doi:10.1186/s40478-022-01422-8)
Supplement: Supplementary file 1 — Additional file 1: Table S1. Clinical Information regarding GBM cell lines. Table S2. Cell markers used for GBM CD105+ cell characterization. Table S3. Differentiation assay of GBM CD105+ cells. Table S4. Panel metrics summary of GBM CD105+ cell exome sequencing. Table S5. Variant calling metrics of GBM CD105+ cell exome sequencing. [file 40478_2022_1422_MOESM1_ESM.pdf]

Table S1 The clinical relative information of the study.

Patient's number	Gender	Age (years)	Pathology	IDH mutant	MGMT methylation	1p19q codeletion	Other profiles	P or R
LU-GBM B1	M	62	GBM	Wild type	No	No	ATRX(+) P53 w+	P
LU-GBM B2	M	62	GBM	Wild type	No	No	NA	P
LU-GBM B3	F	45	GBM	Wild type	No	No	ATRX(+) P53w+	P
LU-GBM B4	M	59	GBM	Wild type	No	No	ATRX(+)	P
LU-GBM B5	F	72	GBM	Wild type	No	No	ATRX(+)	P
LU-GBM B6	M	55	GBM	Wild type	Yes	No	NA	P
LU-GBM B7	F	61	GBM	Wild type	Yes	No	CD34(+) ATRX(+) P53 w(+)	P
LU-GBM B8	M	65	GBM	Wild type	No	No	NA	R
LU-GBM B9	M	57	GBM	Wild type	No	No	KRAS(–) CTNNB1(–) BRAF(–)	R
LU-GBM B10	M	75	GBM	Wild type	Yes	No	ATRX(+)	P
LU-GBM B11	M	41	GBM	Wild type	No	No	NA	P
LU-GBM B12	F	47	GBM	Wild type	No	No	ATRX(+)	P
LU-GBM B13	M	63	GBM	Wild type	No	No	ATRX(+) P53 w+	R
LU-GBM B14	M	55	GBM	Wild type	No	No	NA	R
LU-GBM B15	M	59	GBM	Wild type	Yes	No	NA	P
LU-GBM B16	M	60	GBM	Wild type	Yes	No	NA	P
LU-GBM B17	M	60	GBM	Wild type	No	No	NA	P

LU-GBM B18	F	63	GBM	Wild type	No	No	ATRX(+)	P

M: male, F: female, GBM: glioblastoma, P: primary, R: recurrent.

Table S2 The cell markers using for GBM CD105$^+$ cell characterization.

Antibody name	Supplier	Catalog	Application	Detection methods	Results
CD105	R&D	AF1097	1:100	IF	+
CD105	R&D	AF1320	1:200	IF	+
CD105	BD	560839	5ul/10^6 cells	Flow	+
CD105	R&D	FAB10971P	5ul/10^6 cells	Flow	+
SOX2	Merk	AB5603	1:200	IF	–
CD133	BD	566597	10ul/10^6 cells	Flow	–
NESTIN	Abcam	MAB1259	1:200	IF	+
β III Tubulin	Abcam	ab78078	1:500	IF	+
Vimentin	Dako	GA630	1:500	IF	+
CD31	Dako	M082329	1:100	IF	+
vWF	Abcam	ab9378	1:100	IF	–
CD34	Abcam	ab54208	1:200	IF	+
NG2	R&D	MAB2585	1:200	IF	–
α-SMA	Merk	A5228	1:250	IF	+
PDGF-β	Santa Cruz	SC16569	1:200	IF	+
CD90	Santa Cruz	SC 53116	1:100	IF	–
CD73	Merk	MABD122	1:100	IF	–
FAP	eBioscience	BMS168	1:100	IF	–
Iba1	Abcam	ab5076	1:200	IF	–
TMEM119	Abcam	ab185333	1:100	IF	–
CD11b	Abcam	ab133357	1:200	IF	–
CD68	Biorbyt	Orb13303	1:100	IF	–
CD163	Biorbyt	Orb10343	1:100	IF	–
S100 β	Abcam	ab52642	1:200	IF	–
GFAP	Abcam	ab4674	1:500	IF	–
GFAP	Abcam	ab33922	1:1000	IF	–
NeuN	Abcam	ab104225	1:200	IF	–
PD-L1	R&D	MAB1561	1:100	IF	–
PD-L1	BD	565188	5ul/10^6 cells	Flow	+
Ki67	Abcam	ab16667	1:200	IF	+

IF: Immunofluorescence, Flow: Flow cytometry. "+": antibody-stained positive cells. "–": antibody-stained negative cells.

Table S3 The differentiation functional test for GBM CD105$^+$ cells.

Cell line	Passage of analysis	osteoblast	adipocyte	chondrocytes
LU-GBM B4	P3	+	+	–
LU-GBM B7	P4	+	–	–
LU-GBM B12	P3	+	+	–
LU-GBM B14	P3	+	+	–
LU-GBM B15	P2	+	+	–
LU-GBM B16	P4	+	–	–
LU-GBM B17	P3	+	+	–
LU-GBM B18	P4	+	+	–

"P": Passage. "+" Positive cell staining for hOsteocalcin/ hFABP4/ hAggrecan. "–" Negative cell staining for hOsteocalcin/ hFABP4/ hAggrecan.

Table S4 Panel metrics summary of GBM CD105^{+} cell exome sequencing.

Sample ID	Aligned Reads	Average Coverage	Percentage on target	Q30	Uniformity
LU-GBM B14	366929130 M	485.8x	68.4%	93.9%	95.10
LU-GBM B15	365787418 M	471.4x	72.5%	94.4%	95.11
LU-GBM B16	290568937 M	378.7x	65.7%	93.7%	95.28
LU-GBM B17	234794987 M	305.9x	66.5%	93.8%	95.39
LU-GBM B18	197225790 M	254.7x	66.1%	94.1%	95.33

Panel metrics based on DRAGEN algnment. Target Panel: TWIST-comprehensive. Padding: 20bp

Table S5 Variant calling metrics of GBM CD105^{+} cell exome sequencing.

Sample ID	Variants	Multiallelic	SNP	Ins	Del	Ti/Tv	Het/Hom	Filt SNP	M VC reads
LU-GBM B14	30808	0.3%	92.3%	3.7%	4.0%	2.7	0.6	3.1%	323.7
LU-GBM B15	30106	0.3%	92.2%	3.8%	4.0%	2.7	0.5	3.2%	318.1
LU-GBM B16	35237	0.4%	92.7%	3.5%	3.6%	2.6	1.0	2.4%	253.3
LU-GBM B17	37779	0.4%	92.6%	3.6%	3.7%	2.6	1.6	1.8%	203.3
LU-GBM B18	37946	0.4%	92.7%	3.6%	3.7%	2.6	1.7	1.9%	169.8

Variants: Total number of single-nucleotide polymorphisms (SNPs), Multiple Nucleotide Polymorphisms (MNPs) and structural variations caused by insertions or deletions (INDELs). Multiallelic: The percentage of sites that contains three or more observed alleles. SNP: single-nucleotide polymorphism. Ins: The percentage of insertion. Del: The percentage of deletion. Ti/Tv: The ratio of transition to transtions. Het/Hom: The ratio of heterozygous variant calling or homozygous variant calling. Filt SNP: The percentage of raw SNPs minus the number of passed SNPs. M VC reads: The number of reads used for variant calling, excluding the duplicate reads and the reads outside of target region.
