# Supplementary material for: Glioblastoma CD105+ cells define a SOX2− cancer stem cell-like subpopulation in the pre-invasive niche
Source: Acta Neuropathol Commun. 2022 Aug 29;10:126. doi: 10.1186/s40478-022-01422-8 (PMC9426031; doi:10.1186/s40478-022-01422-8)
Supplement: Supplementary file 3 — Additional file 3. Supplementary Figures: Fig. S1, S2, S3, S4, S6. [file 40478_2022_1422_MOESM3_ESM.pdf]

Supplementary data

Supplementary figures

Fig. S1 GBM CD105$^+$ cell localization and specificity. A CD105$^+$ cells in GBM tumor and pre-invasive niche. Sections stained with CD105 (red) and Ki67 (green). The tumor and peritumor area are verified by Ki67$^+$ cell quantification. ** P<0.01. B The landscape of SOX2$^+$ and CD105$^+$ cells in the GBM preinvasive niche. The images show the distribution of CD105$^+$ and SOX2$^+$ cells by IF. Gamma value = 0.5. C CD105 expression on non-tumor brain tissue and GBM tissue. Non-tumor tissue was collected from the temporal lobe of epilepsy patients (n=3). Immuno-double staining with CD105 and CD31 show differential expression of these markers.

Fig. S2 GBM CD105$^+$ cells cultured in vitro. A The morphology of CD105$^+$ cells cultured for 3 and 30 days. Long-term cell culture of CD105$^+$ cells showing the cell shape changes from tripolar or multipolar cells into flatten and enlarged cells. B Differentiation ability of GBM CD105$^+$ cells. GBM CD105$^+$ cell lines (n=8) were cultured in adipocyte, osteocyte and chondrocyte differentiation conditions for 14 days. Osteocytes and adipocytes were identified by the cell surface markers osteocalcin and FABP4, respectively. C Immunostaining of CD105$^+$ cell with cell type markers in long-term cell culture. Double immunostaining of CD105 (red) and cellular markers (green) of sorted CD105$^+$ cells (under P3). D SOX2$^+$ cells cultured in different concentrations of serum. Ki67 staining (green) shows the difference between each culture condition. Cell proliferation is quantified by Ki67 positive cells. ns P> 0.05

Fig. S3 Kaplan–Meier survival curves comparing different xenograft mouse models. Survival of U87 group (n=5), 2D or 3D cultured CD105$^+$ cell groups (n=14 each group) and mesenchymal stromal cell group (n=5) is analyzed statistically by Log rank test.

Fig. S4 Exome sequencing of GBM CD105$^+$ cells. A The number of locations in the reference genome with the depth of coverage. The depth of coverage of the base position shows the number of high-quality reads mapping to the reference at this position. B Average of the coverage of each chromosome. Average coverage is calculated by the sequencing depth per chromosome. Samples are represented by different color curves. C GC content of sequences. GC content of each sample is calculated by the percentage of sequences. D Mean sequencing quality.

The sequencing quality of each sample is shown by the number of reads with per sequencing quality scores (Phred scores) E The distribution of reads is divided into unique, duplicated and unmapped types. M = million. F The distribution of fragment lengths is estimated by the number of mapped reads. The prevent long flat tail shows the unique points supported by at least 5 reads. The plot is also smoothed down by showing 300 points on the X-axis to reduce noise. G The relations between top 20 mutant genes of GBM $CD105^+$ cells and disease-specific survival in the TCGA database. ns=$P>0.05$

Fig. S6 Cell toxicity assays on $CD105^+$ and SOX^+ cells lines. Different concentration of temozolomide (A, C) and bevacizumab (B, D) cultured with MGMT promotor methylated $CD105^+$ or $SOX2^+$ cell lines (GBM B16) and MGMT wildtype $CD105^+$ or $SOX2^+$cell lines (GBM B14) for 96h. Fluorescence units represent the cell viability detected at 24h, 48h, 72h and 96h. *$P<0.05$

Fig. S1

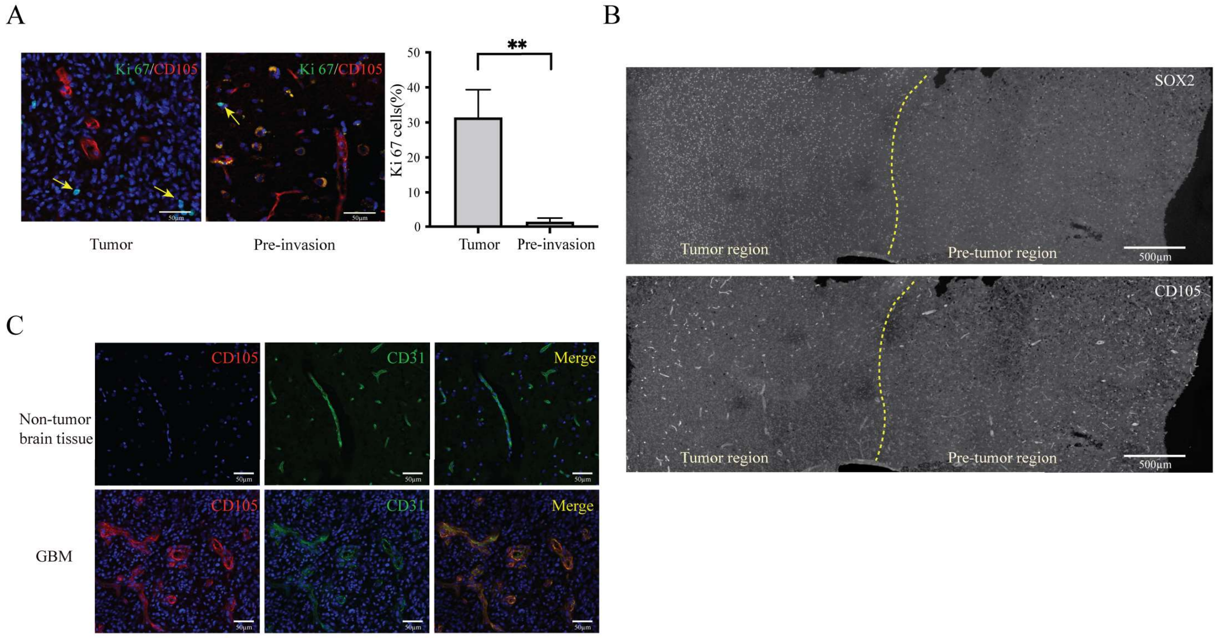
A
Ki 67/CD105
Ki 67/CD105
50μm
50μm
Tumor
Pre-invasion
**
Ki 67 cells(%)
50
40
30
20
10
0
Tumor
Pre-invasion
B
SOX2
Tumor region
Pre-tumor region
500μm
CD105
Tumor region
Pre-tumor region
500μm
C
CD105
CD31
Merge
Non-tumor
brain tissue
GBM
CD105
CD31
Merge

Fig.S2

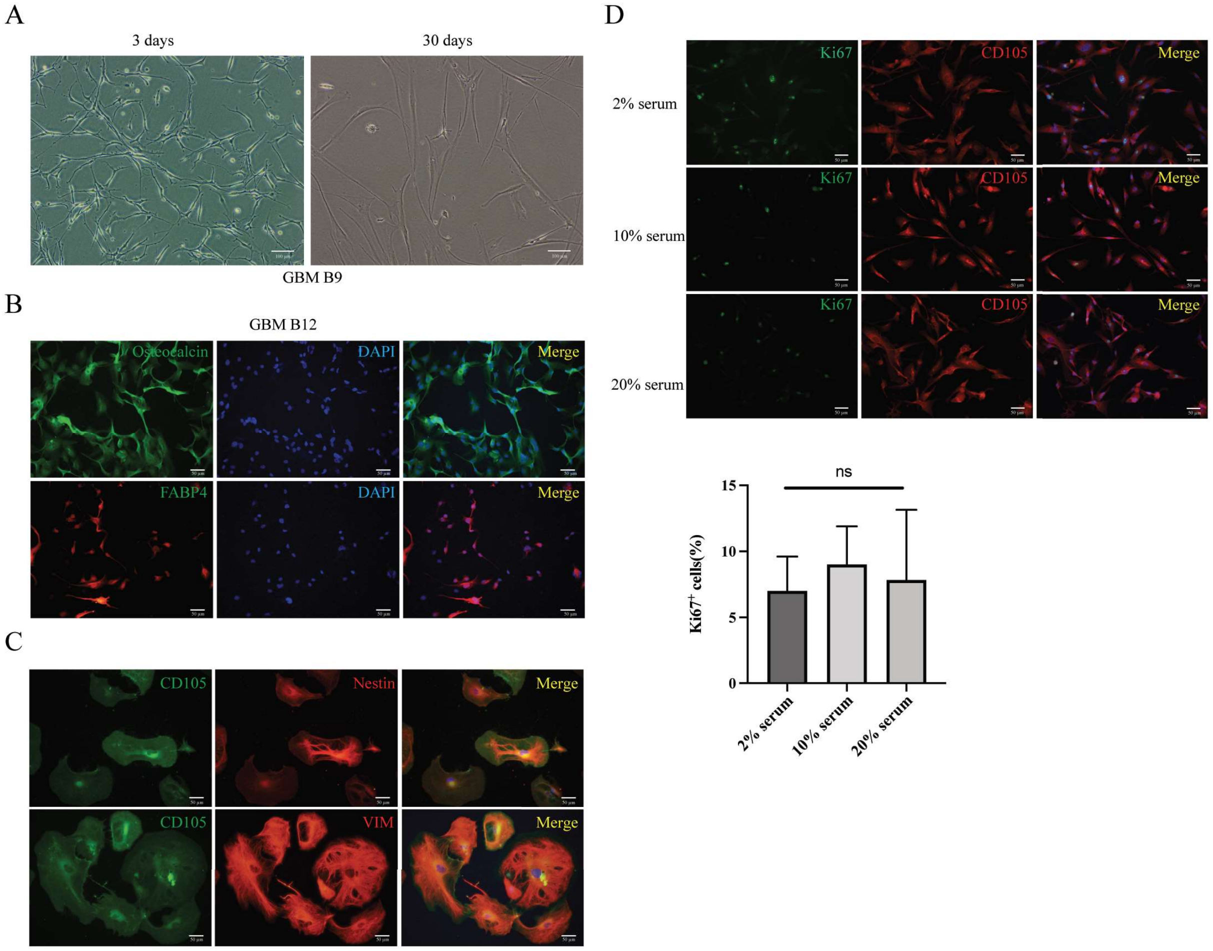
A
3 days
30 days
GBM B9
B
GBM B12
Osteocalcin
DAPI
Merge
FABP4
DAPI
Merge
C
CD105
Nestin
Merge
CD105
VIM
Merge
D
Ki67
CD105
Merge
2% serum
10% serum
20% serum
ns
15
10
5
0
Ki67+ cells(%)
2% serum
10% serum
20% serum

Fig.S3

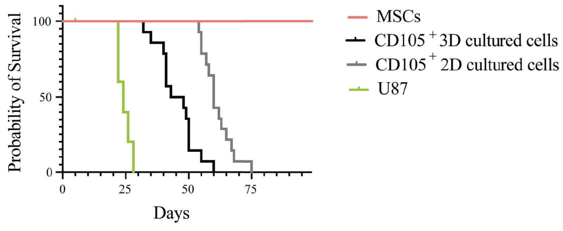
Probability of Survival
100
50
0
0
25
50
75
Days
MSCs
CD105 + 3D cultured cells
CD105 + 2D cultured cells
U87

Fig.S4

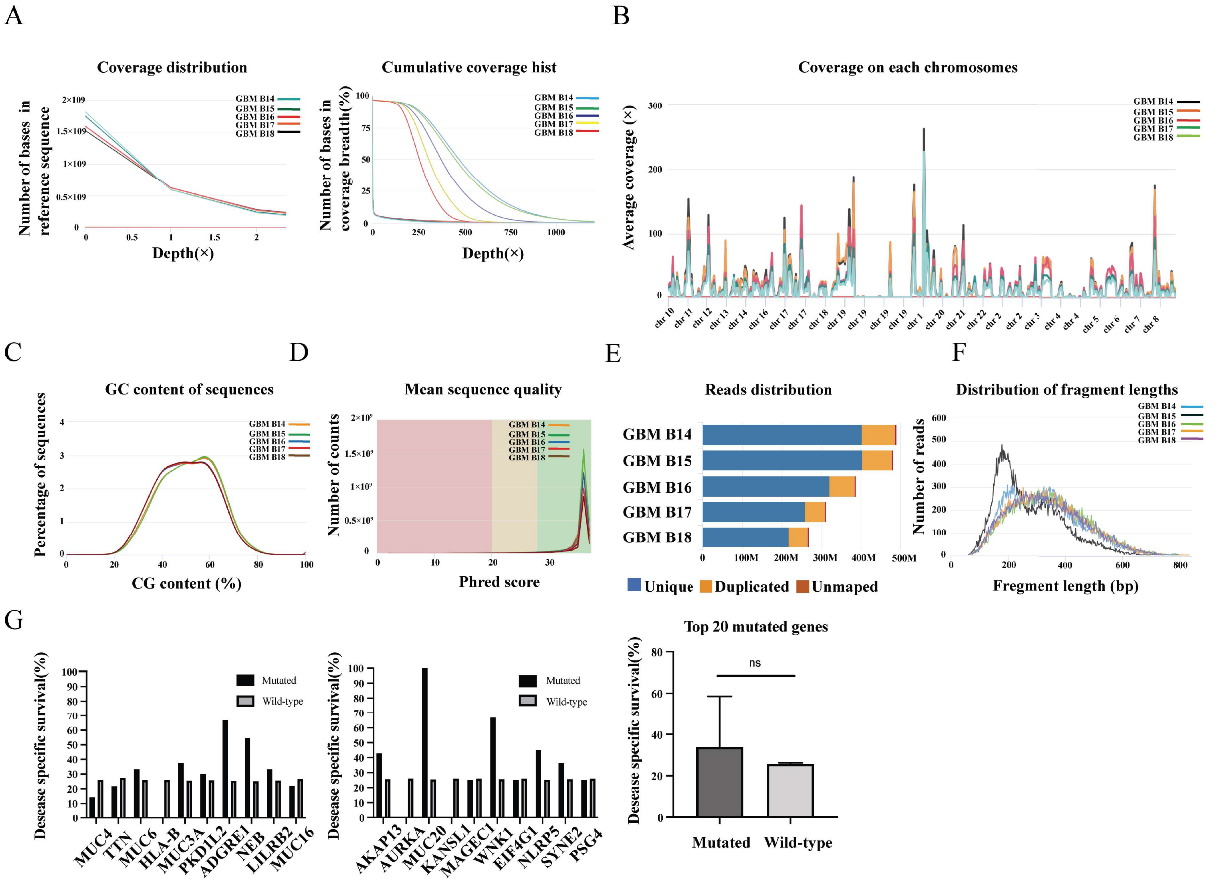

A
Coverage distribution
Cumulative coverage hist
B
Coverage on each chromosomes
C
GC content of sequences
D
Mean sequence quality
E
Reads distribution
F
Distribution of fragment lengths
G
Top 20 mutated genes

Fig.S6

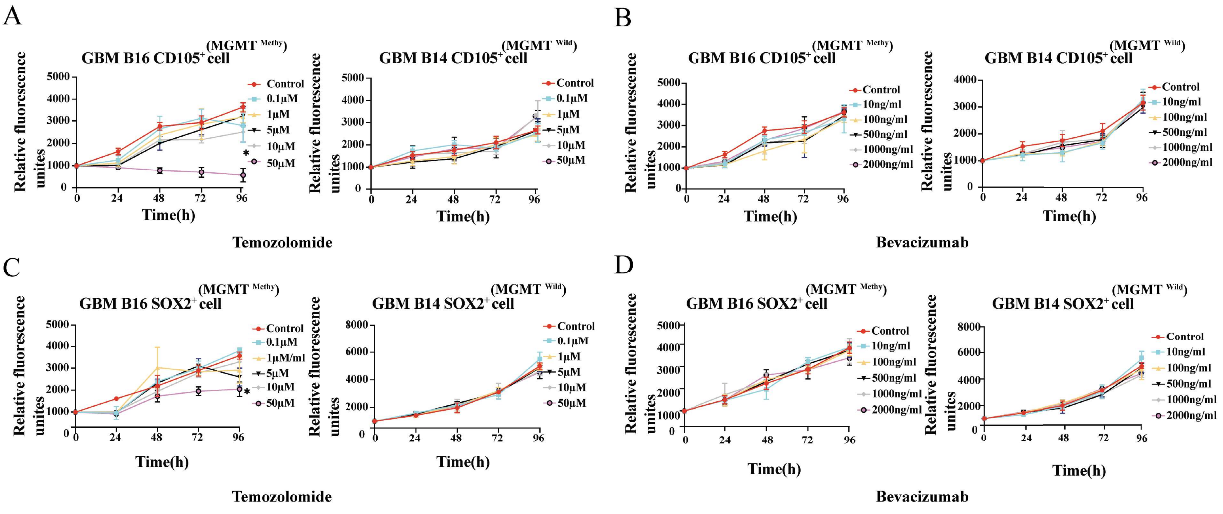

A
GBM B16 CD105+ cell (MGMT Methy)
GBM B14 CD105+ cell (MGMT Wild)
Temozolomide
B
Bevacizumab
C
GBM B16 SOX2+ cell (MGMT Methy)
GBM B14 SOX2+ cell (MGMT Wild)
Temozolomide
D
Bevacizumab
